# The Role of Latin America’s Land and Water Resources for Global Food Security: Environmental Trade-Offs of Future Food Production Pathways

**DOI:** 10.1371/journal.pone.0116733

**Published:** 2015-01-24

**Authors:** Insa Flachsbarth, Bárbara Willaarts, Hua Xie, Gauthier Pitois, Nathaniel D. Mueller, Claudia Ringler, Alberto Garrido

**Affiliations:** 1 Research Centre for the Management of Agricultural and Environmental Risks (CEIGRAM), Department of Agricultural Economics and Social Sciences, Universidad Politécnica de Madrid, Madrid, Spain; 2 Water Observatory, Botin Foundation, Madrid, Spain; 3 Environment and Production Technology Division, International Food Policy Research Institute (IFPRI), Washington, District of Columbia, United States of America; 4 Center for the Environment, Harvard University, Cambridge, Massachusetts, United States of America; Potsdam Institute for Climate Impact Research, GERMANY

## Abstract

One of humanity’s major challenges of the 21st century will be meeting future food demands on an increasingly resource constrained-planet. Global food production will have to rise by 70 percent between 2000 and 2050 to meet effective demand which poses major challenges to food production systems. Doing so without compromising environmental integrity is an even greater challenge. This study looks at the interdependencies between land and water resources, agricultural production and environmental outcomes in Latin America and the Caribbean (LAC), an area of growing importance in international agricultural markets. Special emphasis is given to the role of LAC’s agriculture for (a) global food security and (b) environmental sustainability. We use the International Model for Policy Analysis of Agricultural Commodities and Trade (IMPACT)—a global dynamic partial equilibrium model of the agricultural sector—to run different future production scenarios, and agricultural trade regimes out to 2050, and assess changes in related environmental indicators. Results indicate that further trade liberalization is crucial for improving food security globally, but that it would also lead to more environmental pressures in some regions across Latin America. Contrasting land expansion versus more intensified agriculture shows that productivity improvements are generally superior to agricultural land expansion, from an economic and environmental point of view. Finally, our analysis shows that there are trade-offs between environmental and food security goals for all agricultural development paths.

## Introduction

Latin America and the Caribbean (LAC) globally has the greatest agricultural land and water availability per capita. With 15% of the world’s land area, it receives 29% of global precipitation and has 33% of globally available renewable resources [1]. Large availability of land and water resources fueled rapidly growing exports of primary goods [2, 3]. At the same time, globally, dietary patterns are shifting towards increased consumption of meat and milk products, oils and sugars, and vegetables and fruits, whereas growth of direct human consumption of roots and tubers and grains is either slowing or declining in per capita terms. These dietary shifts are highly resource intensive [4–8]. This increasing global demand pressure gives LAC a pivotal role for meeting global food demands [9, 10]. Over the last 30 years LAC’s agricultural market share has almost doubled from 9.5% in 1980 to 18.1% in 2010 [11]. De Fraiture and Wichelns (2010) [12] and and Hoekstra and Mekonnen (2012) [13] suggest that enhancing agricultural trade leads to natural resource “savings” compared to a world without trade due to global efficiency gains. Thus, trade can play an important role in terms of global food security and environmental efficiency [9] in meeting the estimated 70% increase in global food demand [14]. However, growing food trade requires increasing agricultural production in exporting nations with potential adverse impacts on their natural resource base.

Several studies have evaluated the relationship between trade liberalization and the environment. Some of these studies find a positive impact of more liberal markets on the environment, e.g. [13, 15–18], while others emphasize the negative effects of trade on different environmental indicators, e.g. [17, 19–21]. For example Frankel and Rose (2005) [17] use advanced econometric studies to disentangle the causal relationship between trade liberalization and greenhouse gas (GHG) emissions and other pollutants, and find that trade reduces emissions for most pollutants. On the contrary, Schmitz et al. (2012) [22] conclude that further trade liberalization until 2045 leads to higher economic benefits, at the expense of emitting more CO_2_. Ercin and Hoekstra (2014) [23] compare agricultural water consumption volumes under globalization versus regional self-sufficiency, and find that trade liberalization is only a minor factor in changing water footprints.

The literature focuses on the linkage between agricultural trade liberalization and the environment, but does not specifically distinguish between different possible production systems in exporting regions. There are two dominating views on how to increase agricultural production while minimizing negative environmental impacts, i.e. the so-called land sharing and land sparing argument [24, 25]. The land sharing argument advocates for jointly considering conservation and production objectives on the same land, while the land sparing view supports land specialization with high-yield agriculture coexisting with other areas devoted to nature conservation. Promoting a land sharing strategy requires extensification of agricultural production as agricultural inputs on farm decrease. This could increase the agricultural land footprint to keep up with production levels. As agricultural land footprints increase, the risk of deforestation and land clearing also rises. This in turn might threaten biodiversity and lead to GHG emissions [26, 27]. A land sparing approach on the contrary will require further intensification of agriculture to increase average yields per hectare (ha). Yield improvements depend on the adoption of various conventional and agro-ecological management practices, including the use of high-yielding cultivars, and enhanced management practices to reduce abiotic and biotic plant stresses. [28]. In this study we implement land sparing through the following management practices: (1) supplemental water through irrigation (or rainwater harvesting) and (2) supplemental nutrients through additional fertilizers. However, overexploitation of water resources and climate change make it difficult to further expand irrigation in some areas [29, 30]. Furthermore, fertilizer use can lead to water and soil pollution, causing negative impacts on freshwater and terrestrial ecosystems [26]. In order to respond to these pressures, finding ways for “sustainable intensification” has become central. This could mean increasing yields on underperforming landscapes while reducing adverse environmental impacts of agricultural systems [31, 32]. Some studies focus on closing yield gaps by optimizing management practices [26, 31, 33, 34], usually using static methods referring to just one point in time. Others focus on combining supply and demand side measures, e.g. by applying traditional and modern breeding techniques to improve yields, while emphasizing the need to limit food waste and over-consumption [32, 35].

Our study aims at investigating the role of LAC’s agriculture for global food security and associated environmental trade-offs of contrasting scenarios of agricultural production out to 2050. Specifically, we explore changes in water footprints and water quality, as well as impacts on biodiversity and carbon stocks from land use change by 2050 from alternative agricultural production pathways and identify related environmental hotspots. We focus on LAC because the region has become one of the main food producers globally and is likely to continue on this trajectory under further agricultural market liberalizations.

## Materials and Methods

### Scenarios

In our modeling exercise, we contrast five alternative agricultural development pathways with a Business-as-Usual (BAU) scenario, which reflects what we believe are most likely changes in key human and agricultural development parameters out to 2050. Note that while assumptions under the BAU scenario are applied globally, alternative future scenarios focus on LAC. The BAU scenario (1) uses the UN medium variant projections with respect to population growth [36]. The economic growth assumptions are based on the TechnoGarden scenario of the Millennium Ecosystem Assessment [37]. BAU assumes climate change based on the “A1B” scenario specified in the Special Report on Emissions Scenarios (SRES) of the Intergovernmental Panel on Climate Change [38]. We apply different global climate models (GCMs) as climate inputs to the International Model for Policy Analysis of Agricultural Commodities and Trade (IMPACT). (IMPACT model details are described in the following section.) However, we only present results from MIROC model runs, because results between different GCMs do not deviate much. (For details on the sensitivity between different GCMs see S1 Text). The BAU scenario assumes a continuation of past trends in irrigated and rainfed area growth rates as well as crop and livestock productivity growth rates with a gradual slow down in growth. Current trade policies are kept constant over time, so no further trade liberalization is assumed. Details on the BAU assumptions and values used in the base year can be found in Nelson et al. (2010) [30].

The following alternative future scenarios are analyzed: (1a) A global liberalized trade scenario, (2) a LAC intensification scenario, (3) a LAC sustainable intensification scenario, (4) a LAC closed yield gaps scenario and (5) a LAC extensification scenario. The distinct features of each scenario are summarized in Table 1. The numbers should be interpreted as the deviation from the BAU scenario. The selection of parameters is described in further details below. Note that due to high agricultural specialization in LAC, we focus our analysis on those food crops that together accounted for more than 70% of agricultural production in 2010: maize, rice, wheat, soybeans, sugarcane, potatoes and sorghum. Livestock products included in the study are cows, sheep, goats, pigs and chickens. However, for the pasture land estimations only cows, sheep and goats are considered (for details see S2 Text).

**Table 1:** Alternative future scenarios for 2010 to 2050 (changes compared to BAU (1)).

Parameters	(1a) BAU liberal	(2) Intensification	(3a) sustainable intensification (higher nutrient use efficiency)	(3b) sustainable intensification (precision agriculture)	(4) Closing yield gaps	(5) Extensification
Livestock number growth	n.c.	n.c.	n.c.	n.c.	n.c.	+ 30% (LAC)
Livestock yield growth	n.c.	+ 30% (LAC)	+ 30% (LAC)	+ 30% (LAC)	+ 30% (LAC)	- 30% (LAC)
Yield growth changes of seven food crops*	n.c.	+ 60% (LAC)	+ 60% (LAC)	+ 60% (LAC)	closed yield gaps, region and crop specific (LAC, gradually until 2050)	- 60% (LAC)
Irrigated area growth seven food crops*	n.c.	+ 25% (LAC)	+ 25% (LAC)	+ 25% (LAC)	+ 25% (LAC)	- 25% (LAC)
Rainfed area growth seven food crops*	n.c.	Zero exogenous area growth (LAC)	Zero exogenous area growth (LAC)	Zero exogenous area growth (LAC)	Zero exogenous area growth (LAC)	+ 15% (LAC)
Basin efficiency (ratio between 0 and 1)	n.c.	n.c.	+ 15%-points (gradually until 2050)	+ 15%-points (gradually until 2050)	n.c.	n.c.
Increased nutrient use efficiency (NUE)*	n.c.	n.c.	increased NUE by 20% (LAC, in 2050)	n.c.	n.c.	n.c.
Precision Agriculture*	n.c.	n.c.	n.c.	optimized nitrogen use (LAC, in 2050)	n.c.	n.c.
Trade distortions	- 40% (globally, gradually until 2050)	- 40% (globally, gradually until 2050)	- 40% (globally, gradually until 2050)	- 40% (globally, gradually until 2050)	- 40% (globally, gradually until 2050)	- 40% (globally, gradually until 2050)

Note: n.c. = no change compared to Business-as-Usual (BAU) assumptions (for base year details see Nelson et al. (2010) [30], * = applied to maize, rice, wheat, soybeans, sugarcane, potatoes, sorghum. LAC means that changes are applied to Latin America and the Caribbean, Globally means that changes compared to BAU are applied globally.

The liberalized trade scenario (1a) uses the same assumptions as BAU, but with globally gradually liberalizing trade regimes, following an historically derived pathway. Based on the literature, we implemented a 10% trade barrier reduction in each decade starting 2010 until 2050 [22, 39, 40].

The intensification scenario (2) and the sustainable intensification scenario (3) deviate from the BAU scenario following the high-AKST (Agricultural Knowledge and Science and Technology) scenarios of the IAASTD report [41]. Here, we assume more agricultural research and development (R&D) in the future, and therefore higher crop and livestock yield growth as well as expansion of irrigation. This scenario also assumes no additional growth in rainfed area to simulate land use policies that promote land sparing. Note that even under the assumption of zero exogenous rainfed area growth, growth of rainfed area can still be triggered by price increases, because farmers’ production decisions depend on prices. The sustainable intensification scenario (3) further assumes improved basin water use efficiencies through advanced irrigation technologies and sound management in those regions in LAC that suffer from water stress (affected spatial units are listed in S3 Text). Compared to the BAU scenario, basin efficiency is 15 percentage points higher by 2050 in those spatial units. The sustainable intensification scenario is further sub-divided into (3a) and (3b) to explore environmental impacts of two alternative agricultural technologies. Both, assume high yield growth rates, but at lower N-emission rates due to optimized fertilization and plant uptake. Under (3a) higher nutrient use efficiencies (NUE), expressed in crop yield per kg nutrient applied, are considered and under (3b) precision agriculture is assumed to be widely in use.

Scenario (4) on closing yield gaps follows the same assumptions as the intensification scenario (2), but crop yield growth rates are increased according to existing yield gaps, instead of the high-AKST growth rates. Yield gaps could be closed by improved management practices or through accelerated technological change. The yield gap is defined as the difference between observed yields and potentially attainable yields. We close 75% of the yield gap as closing 100% is unlikely to be economically sensible. We also conducted a sensitivity analysis where we close 100% of the yield gap. However, the main food security and environmental results do not change substantially. Therefore, we do not report results from the sensitivity analysis. We follow the approach of Mueller et al. (2012) [31] and identify attainable yields for each crop in each low-yielding area in LAC by matching them to the corresponding high-yielding world areas within zones of similar climates. For a detailed description of the methodology see Mueller et al. (2012) [31]. Our modeling exercise (described below) is conducted at the subnational level. To obtain attainable yields per crop at this spatial resolution in LAC, we aggregate the high-yield information of different climate zones within each spatial unit (available at a 0.5 by 0.5 degree longitude-latitude grid resolution). Weights are chosen to reflect the share of area harvested in each climate zone within one spatial unit. Some initially low-yielding areas can close yield gaps under the BAU scenario (1) already. In scenario (4) we only increase yield growth rates compared to BAU growth rates in those regions where BAU yield growth rates were not sufficiently high to close yield gaps by 2050. For the other regions, BAU growth rates without additional yield growth are assumed. (Areas with remaining yield gaps (area abbreviations are explained in S1 Table) are listed in S2 Table.)

We contrast the described intensive scenarios (2/3a/3b/4) to a scenario that assumes more extensive agricultural practices in the future. Specifically, scenario (5) assumes lower crop and livestock yield growth and less expansion of irrigation, but instead higher rainfed area growth rates and accelerated livestock numbers’ growth. The values are chosen following the low-AKST scenario of the IAASTD report [41].

Note that all scenarios, except for the BAU scenario assume further trade liberalization. Scenario (1a) serves to isolate the effects of trade liberalization from those of choosing the different production systems described in scenarios (2) to (5).

### Modeling framework

Here, we give a brief overview of the methodology applied in the study. A more detailed methodology description as well as all relevant model equations for the study can be found in the Supporting Information.

**The IMPACT model**. We use IMPACT to analyze the above described scenarios. A complete mathematical description of the model can be found in Rosegrant (2012) [42]. IMPACT is a global multi-market, dynamic partial-equilibrium model of the agricultural sector that provides long-term projections of global food supply, demand, trade, prices, and food security developed by the International Food Policy Research Institute (IFPRI). It covers 46 agricultural commodities, including all cereals, soybeans, roots and tubers, meats, milk, eggs, oils, meals, vegetables, fruits, sugar and sweeteners, and other foods. Dietary changes are taken into account by adjusting demand elasticities to accommodate the gradual shift in demand from staples to higher value commodities like meat and milk products, especially in developing countries. This assumption is based on expected economic growth, increased urbanization, and continued commercialization of the agricultural sector.

As described in the scenarios, the focus of this study lies on modeling trade liberalization and changes in supply side factors, developed to analyze LAC’s contribution to meet higher future calorie demand. The alternative scenarios (1a, 2–5) assume trade liberalization by a gradual reduction of producer support estimates (PSE), consumer support estimates (CSE) and marketing margins (MI) globally until they are 40% lower in 2050 compared to 2010 levels. The different future agricultural production pathways are simulated by adjusting BAU growth rate assumptions in LAC according to the scenario specific assumptions. The parameters affected are exogenous crop and livestock yield shifters, exogenous rainfed and irrigated area shifters, and exogenous livestock number shifters embedded in a set of equations. Apart from these exogenous factors, crop and livestock specific yields, area harvested and the number of slaughtered animals react endogenously to price movements. Furthermore, we account for the biophysical effects from climate change in all world regions. The Decision Support System for Agrotechnology Transfer (DSSAT) uses changes in precipitation and temperature to model climate change productivity effects by calculating location specific yields in different years, and converting these to a growth rate which is then used as a yield shifter. For details on how climate change is modeled in IMPACT see Nelson et al. (2010) [30]. Water stress (sometimes aggravated by climate change) is captured as part of a loosely coupled hydrology model which provides gridded output of hydrological fluxes, namely effective rainfall, potential and actual evapotranspiration, and runoff. These parameters are in turn used as inputs to a Water Simulation Model (WSM) that balances water availability and uses within various economic sectors, at the global and regional scale. Globally, IMPACT uses a disaggregation of 280 spatial units, from now on called “food-producing units” (FPU), which represent the spatial intersection of 115 economic regions and 126 river basins. For LAC, IMPACT includes 31 FPUs which are illustrated in S1 Fig. and S1 Table. (For a more detailed IMPACT model description with the model equations relevant for our study see S4 Text.)

Firstly, we use the IMPACT model to simulate scenario-specific 2050 changes of global agricultural trade flows, international food prices, and the total number of malnourished preschool children (under five years old) in different world regions. The number of malnourished children serve as an indicator for food security in our analysis. The relationship used to estimate this food security indicator is based on a cross-country regression model developed by Smith and Haddad (2000) [43]. (For more details on the food security estimation see S5 Text.) Note that all variables used for the malnutrition regression (see S5 Text) are assumed to be exogenous, and IMPACT results only alter calorie availability due to changes in supply and demand and resulting world prices. This is a somewhat simplistic assumption as different trade assumptions might also influence some of the other variables. However, regional and global trends are well reflected in this structure.

Secondly, as an advantage over many other economic models, IMPACT treats future water demand and supply endogenously, responding to sectors providing and demanding water. We use this feature in order to calculate water footprints of agricultural production and identify water scarcity hotspots under alternative production systems. More specifically, we differentiate between the green water footprint of production (GWF) and the blue water footprint of production (BWF). The GWF is defined as the rainwater evaporated or incorporated into a specific crop by FPU, while the BWF reflects the volume of surface or groundwater evaporated or incorporated into a specific crop or livestock in an FPU [44]. (For details on how GWF and BWF are calculated see S6 Text.) Irrigation water scarcity is analyzed with a water stress index, which measures the gap of water supply and demand in each FPU. In stressed FPUs, with a water stress index below one, water supply cannot meet crop demands, leading to yield reductions.

An IMPACT model validation (with respect to area harvested which is one major variable used in our study) is illustrated in S2 Fig.

**Water quality assessment**. Impacts of expanded or intensified agricultural activity on the water environment are assessed by quantifying variations of nitrogen-based pollutants over time under each of the scenarios considered. This involves linking IMPACT results to the Soil and Water Assessment Tool (SWAT). SWAT is a physically-based watershed model equipped with functions to simulate the main processes of nitrogen cycles in agricultural river basins [45]. The model has been extensively applied to investigate water quality issues related to agricultural nitrogen emissions (N-emissions), e.g. [46–48]. In this study, we parameterize the SWAT model on a 0.5 by 0.5 degree longitude-latitude grid to estimate annual rates of agricultural N-emissions (including emissions from both crop and pasture land) across different FPUs in LAC according to determined nitrogen input rates on agricultural land and climate conditions in the base year and under future scenarios. The term N-emissions refers to the discharge of particulate and dissolved nitrogen-based pollutants from land to water environments. In addition to estimating the effects of more or less intensified agricultural production systems, we constructed two sustainable intensification scenarios (3a/3b). Under the sustainable intensification scenario with NUE improvement (3a), input rates of fertilizer and manure nitrogen on crop land are adjusted to mimic NUE enhancement by +20%. To represent precision agriculture techniques in the sustainable intensification scenario (3b), we invoke an auto-fertilization function in the SWAT model [49] to determine the quantity and timing of nitrogen fertilizer/manure applications, given nitrogen requirements of the major crops. A more detailed methodological description can be found in S7 Text.

**Carbon assessment**. We quantify the impacts on carbon stock losses linked to the projected expansion of cropland and pasture areas due to livestock production in LAC between 2010 and 2050 for each of the agricultural production scenarios. Land use dynamics are complex and the link between agricultural expansion and deforestation in LAC is not straightforward, i.e. many cropland areas are now expanding on existing pastures, but indirectly such expansion is pushing the agricultural frontier beyond as cattle ranching activities are displaced [50, 51]. Since IMPACT does not provide information on the likely land use transitions, our carbon (C) impact estimations assume the following alternative land use pathways: (i) all new cropland area expands over former natural vegetation; or (ii) all new cropland area expands over existing pastures. Estimating carbon stock changes for these different land use pathways provides us with a lower and upper bound estimation of carbon storage losses linked to cropland expansion. For livestock production, we assume that all future pasture expansion will expand over former natural vegetation. In those FPUs where a reduction in the agricultural area is expected, we assume that the new abandoned agricultural areas are able to restore their C stocks back to their original values i.e. those of the original natural vegetation, due to natural succession and forest regrowth.

To calculate the carbon trade-offs we first estimate aboveground and belowground carbon contents of natural vegetation, pastures and croplands of the seven different crops considered in this study by FPU. Then, we estimate the changes in carbon stocks resulting from the conversion of (1) natural vegetation to land dedicated to each of the seven crops, (2) natural vegetation to pastures, and (3) pastures to land of each crop type, by FPU and under each of the different scenarios of agricultural production. For a detailed description of the calculation of the projected crop and pasture area up to 2050 we refer to S2 Text. Please see S8 Text for a complete description on the data and methodology used to estimate carbon stocks and changes.

**Biodiversity assessment**. We use species-area relationships (SAR) to account for potential biodiversity trade-offs associated with each of the scenarios of agricultural production in LAC. Specifically, we apply a countryside model [52] to predict changes in endemic bird’s risk of extinction and endangerment (expressed as an index in %) associated with the projected increase in cropland and pasture area between 2010 and 2050. We limit the study to birds since taxon’s sensitivity to different forms of land use change is well studied, and data on their conservation status and spatial range are most reliable, updated, and available. To avoid the scale dependency factor [53] when assessing the extinction and endangerment rate we limit our study to endemic birds i.e., species with breeding range limited to LAC region.

As with the carbon assessment, the birds’ risk of extinction and endangerment due to cropland expansion is assessed by taking into account the different land use pathways to obtain a lower and upper bound of biodiversity trade-offs. As for pastures, the risk index is estimated assuming that future pasture areas will expand over former natural vegetation. Again, we account for agricultural abandonment and forest regrowth when estimating the total impacts on biodiversity.

To assess the bird’s risk of extinction and endangerment by FPU and under the different scenarios we estimated: (i) the actual number of birds and the percentage of threatened species by FPU; (ii) the area of the main land uses per FPU (natural vegetation, pastures, cropland, urban/artificial); and (iii) the linear relationship between the percentage of threatened species and habitat availability and suitability. A detailed description of the country-side model and data used can be found in S9 Text.

## Results

### Global food security in 2050

With increasing globalization, population growth and dietary changes, LAC will likely supply even larger amounts of food to the rest of the world by 2050. We find that, depending on the production scenario, LAC can further strengthen its net export position for some of the seven crops and the livestock products investigated in this study. Although for some staple crops, like maize and potatoes, markets will still be dominated by North America and Europe under all scenarios in 2050, LAC will grow production and by 2050 become a maize and potato net exporter. Under BAU liberal (1a), as well as under the intensification scenarios (2/3) this trend is even more pronounced. This means that no substantial additional land for these crops will be required if more irrigation is applied and rainfed and irrigated yield improvements are sufficiently high. The same holds for the closing yield gaps scenario (4) for some crops. Under BAU (1), yields gaps in potato production remain pronounced up to 2050. Therefore, gradually closing those gaps between 2010 and 2050 would lead to a stronger market position. For maize, yield gaps in LAC are rather small, so net exports are even smaller than under BAU (1), because we assume a much slower rainfed area growth without substantial improvements in yield growth rates. The extensification scenario (5) assumes increased rainfed area growth, and slowing irrigated area expansion rates and yield growth rates. The results show that only increasing rainfed area cannot compensate for the productivity slowdown and it even eliminates the positive effects of trade liberalization. LAC’s strongest agricultural export products are soybeans and sugar with sugar made of sugarcane. For both products, world trading volumes increase substantially until 2050, with trade liberalization reinforcing LAC’s net export position. For soybeans, however, intensifying production without allowing for rainfed area growth (scenarios (2/3/4)) reduces LAC’s net export position compared to BAU (1). This can be explained by the fact that soybeans are largely produced under rainfed conditions, and further production expansion without area expansion is rather difficult. In the case of sugarcane, intensification (2/3) augments net sugar exports. Also for beef, market liberalization combined with the assumption of accelerated yield growth under scenarios (2), (3) and (4) strongly reinforce LAC’s net export position. Growing livestock numbers without substantial yield improvements, as assumed under the extensification secenario (5), reduces LAC’s comparative advantage in beef production compared to BAU liberal (1a), but net exports are still higher than under BAU (1). For wheat, rice and sorghum LAC remains at a net importing position, regardless of the scenario assumed for the future.

**Fig 1:**
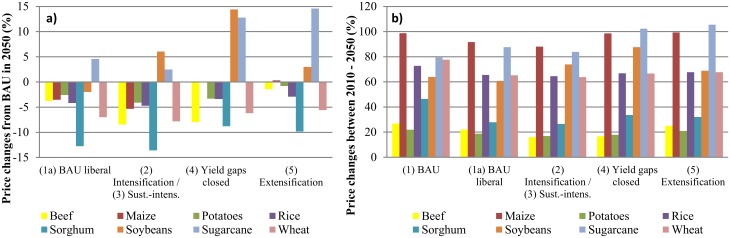
World food price changes under different scenarios. (a): World price deviations in percent compared to BAU (1) in year 2050; (b): World price changes in percent within each scenario from 2010 to 2050. BAU refers to the Business-as-Usual scenario. Scenarios are described in Table 1. The intensification (2) and sustainable intensification (3) scenarios are presented together, because both scenarios have the same productivity assumptions and they only differ in terms of of natural resource efficiencies. Thus, the implications for agricultural markets are the same under both scenarios.

Fig. 1a shows the percent change in world prices of each scenario from the BAU (1) price level in 2050. From “BAU liberal” (1a) it is clear that trade liberalization itself has the strongest effect on prices. This is due, firstly, because liberalization is implemented globally, and secondly changes in yield, livestock numbers and area growth rates are only assumed for LAC. If additional productivity improvements were implemented globally, the effects on world production, and in turn on prices, would likely be much more pronounced. In Fig. 1b we see that real world prices will increase in all scenarios between 2010 and 2050, with the steepest increases for almost all products under BAU (1). One exception is sugar whose price would increase more under all alternative scenarios than under BAU (1). Sugar is one of the world’s most highly protected agricultural commodities [54], thus reducing market distortions leads to shifts in production and consumption, and consequently to higher world prices. Intensifying production of sugarcane or closing yield gaps (scenarios (2/3/4)) can attenuate this effect, while extensification (scenario 5) further exacerbates pressure on sugar prices. In contrast to sugar, trade liberalization (scenario 1a) has price reducing effects for the other six crops and beef. This also holds for soybean prices. However, the price increase of soybeans between 2010 and 2050 cannot be reduced through extensification (scenario 5), or intensification (scenarios 2/3) or closing yield gaps (scenario 4). The extensification scenario (5) assumes 60% slower yield growth rates due to lower agricultural inputs which cannot be compensated by the 15% accelerated rainfed area growth. The intensification scenarios (2/3) and, to a much lesser extent, the closing yield gaps scenario (4), assume accelerated yield growth rates, but no further exogenous rainfed area growth. Since soybeans are mostly produced under rainfed conditions, the assumed zero future rainfed area growth leads to higher world prices compared to a situation with faster area growth. Thus, to further augment soybean production, trade liberalization, accelerated yield improvements, and allowing for rainfed area expansion seem to be equally important. For all other crops (wheat, maize, sorghum, potatoes, rice) the intensification scenarios (2/3), and for potatoes the closing yield gaps scenario (4), reinforce the price reducing effect of trade liberalization. On the contrary, allowing for more rainfed area growth, but reducing productivity growth (scenario 5) does not show positive effects. In general, the yield gaps scenario (4) has rather limited production effects, because for most crops (except for potatoes), many areas in LAC are already among the world’s high-yield areas.

**Fig 2:**
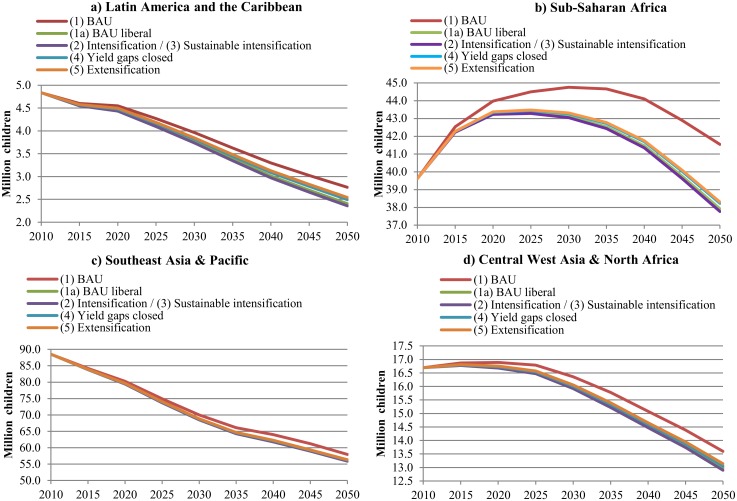
Number of malnourished children from 2010 to 2050 associated with different future scenarios. BAU refers to the Business-as-Usual scenario. Scenarios are described in Table 1. The intensification (2) and sustainable intensification (3) scenarios are presented together, because both scenarios have the same productivity assumptions and they only differ in terms of of natural resource efficiencies. Thus, the implications for agricultural markets are the same under both scenarios.

Changes in global food supply and food prices affect people’s ability to access food across the world, particularly in developing countries. Hence, different production conditions in LAC and the trend towards more open food trade will have effects on future food security globally. Fig. 2 illustrates that the number of malnourished children is projected to decrease by 2050, though less under BAU assumptions (scenario 1) and the most under the intensification scenarios (2/3). Trade liberalization (scenario 1a) has positive effects on calories availability, reducing food insecurity. Intensifying food production (scenarios 2/3) in LAC reinforces the positive effect of trade liberalization slightly. However, the largest improvements in food security are achieved by assuming trade liberalization, because all countries benefit whereas the accelerated productivity gains are focused on the LAC region only. In addition, soybeans and sugarcane are not only food crops, but also used for the production of feedstuff or biofuel. Beef is often a luxury product consumed by richer segments of the world population. Therefore, the relevance of these products for improving food security is somewhat more limited. Moreover, we see that food security in LAC (Fig. 2a) and in the Southeast Asia & Pacific region (Fig. 2c) improves fast with a linear trend, while Central-West Asia (Fig. 2d) and Sub-Saharan Africa (Fig. 2b) show less improvements. This can be explained by the fact that Sub-Saharan Africa is expected to experience very rapid population growth without concomitant food production growth, and continued lack of access to safe water as well as continued limited improvement in female secondary education, key variables limiting food security gains (see S5 Text for details on these variables). Therefore, these socio-economic factors seem to be very dominant in explaining food insecurity. However, we also see that the region would especially benefit from more liberal agricultural markets, despite its remaining difficulties.

### Environmental trade-offs

This section describes the different environmental impacts of our different future production pathways. Results for environmental indicators include a temporal scale and a spatial scale. This means that we show changes between 2000 and 2050, and also differences between LAC’s regions in 2050 to highlight environmental hotspots.

**Fig 3:**
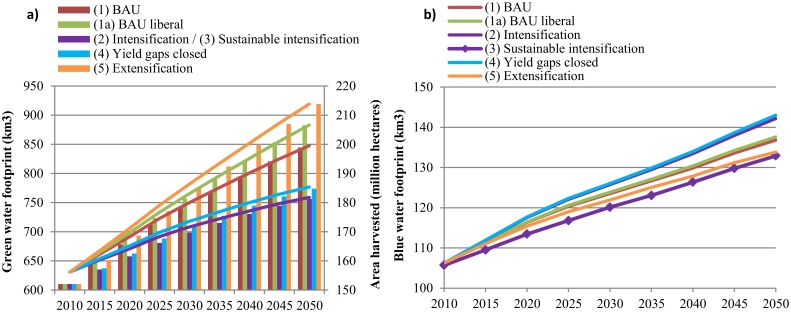
Green and Blue Water Footprints under different scenarios in Latin America and the Caribbean from 2010 to 2050. (a): Evolution of the total Green Water Footprint (represented as the line chart on the left axis) and area harvested (represented as the bar chart on the right axis) of all crops in Latin America and the Caribbean from 2010 to 2050. (b): Evolution of the total Blue Water Footprint of all crops and livestock in Latin America and the Caribbean from 2010 to 2050. BAU refers to the Business-as-Usual scenario. Scenarios are described in Table 1. For green water, the intensification (2) and sustainable intensification (3) scenarios are presented together, because both scenarios have the same productivity assumptions and they only differ in terms of blue water use.

**Impacts on water resources**. From an environmental perspective, it is crucial to establish whether the water used in agriculture originates from rainwater lost in evapotranspiration and evaporation during the production process (green water) or from surface and/or groundwater sources (blue water). It has been argued that the use of green water in crop production is considered more sustainable than blue water use, although this is not necessarily the case if either blue water resources are sustainably managed [55] or expanding rainfed agriculture is associated with massive land clearing and deforestation. Due to its favorable climate, most of LAC’s agriculture is rainfed. The lines in Fig. 3a show how the GWF evolves over time in LAC under the different future scenarios. The GWF increases overall, although the increase is smaller under those scenarios in which intensification is greater. As expected, extensifying (5) food production leads to the highest increase in green water use, followed by the trade liberalization scenario(1a) which does not consider accelerated yield growth. An expansion of rainfed agricultural production is usually associated with an increase in area harvested, because the potential for productivity improvements is lower than for irrigated agriculture. The bars in Fig. 3a confirm this relationship. This means that the environmental costs are rather related to land conversion than to water. These trade-offs will be discussed below in the sections related to carbon stock losses and risk of biodiversity losses.

In contrast to land expansion, agricultural production can also increase by expanding irrigation. Expanding irrigated area directly affects water resources. Fig. 3b shows the BWF over time for all scenarios. Liberalizing trade slightly increases the BWF from crop and livestock production. The highest blue water use is associated with scenarios (2) and (4) demonstrating the trade-offs between achieving higher yields and conserving water. As expected, extensifying agriculture (5) would save blue water compared to the BAU scenario (1). The sustainable intensification scenario (3) reduces the BWF even more while maintaining high yields. This highlights the importance of investments in water saving technologies and adequate water management practices. Note that even though productivity and area changes are only implemented for the seven major crops, water footprints per FPU are calculated for all IMPACT commodities. We do this, because in some regions fruits and vegetables, cotton or even livestock are the main water consumers rather than the seven crops studied in detail for LAC. Furthermore, improved basin efficiencies are introduced at the river basin scale and thus apply to all IMPACT crops.

Since LAC as a region is rather water abundant, increasing blue water use would not necessarily lead to unsustainable extraction rates. However, in some water scarce areas, expanding irrigated area would exacerbate water stress. S3 Table lists those FPUs in LAC where irrigation demand cannot be fully met. In most water stressed FPUs water scarcity increases over time, with the exception of the Caribbean (CAR_CCA) and Yucatan (YUC_CCA) in Central-America, and Uruguay (URU_URU)as well as Brazil at the border to Uruguay (URU_BRA) in South America. Here (effective) precipitation increases over time, leading to an increasing ratio of water supply over demand. A water-stressed hotspot in Central America is Cuba (CUB_CCA), while in South America Tocantins in Brazil (TOC_BRA) and Coastal Peru (PEC_PER) rank among the most water stressed regions. In all water scarce regions, irrigation supplies suffer the most under the more intensive scenarios (2) and (4), followed by the BAU scenarios (1) and (1a). Again we see that extensifying agricultural production systems (5) would slightly alleviate stress on water resources. Pressure is lowest under the sustainable intensification scenario (3).

This shows that increasing irrigation should go hand in hand with better water management and technologies and adequate irrigation practices. A large share of irrigated areas is subject to both land and water degradation as a result of poor irrigation management, eventually affecting crop yields and long-term productivity of the land. In LAC, additionally, water stress also emanates from its large urbanized centers. Right now 23% of LAC’s population (125 million people) live in water scarce basins [10].

**Fig 4:**
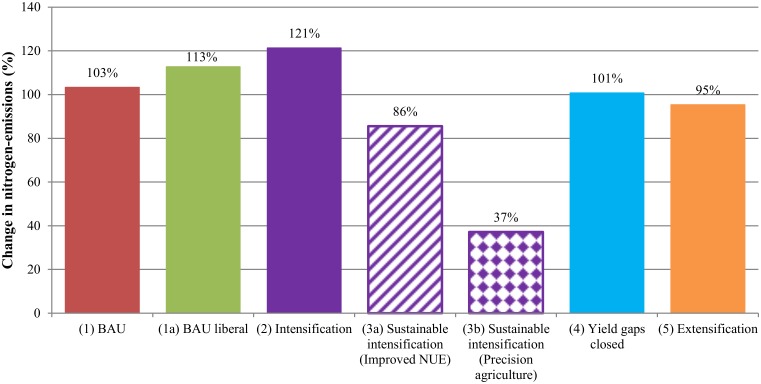
Changes in nitrogen-emission rates in Latin America and the Caribbean between the base year 2000 and 2050 (in%). BAU refers to the Business-as-Usual scenario. Scenarios are described in Table 1.

**Water quality impacts**. Excessive amounts of nitrogen, together with excess phosphorus, in water bodies often cause eutrophication. Agriculture is a major human source of N-emissions. For Latin America as a whole, the risk of water quality degradation due to N-emissions increases until 2050, irrespectively of the future production scenario considered (see Fig. 4). Under BAU assumptions (1), total N-emissions will increase by about 103% in 2050. If trade is liberalized (1a), the increase will be even larger, at around 113%. As expected, the extensification scenario (5) shows a relief on N-emissions due to the assumption of a slower increase in fertilizer application rates. In contrary, the intensification scenario (2) would lead to the highest risk of water quality degradation, because accelerated yield growth will in part be achieved by higher N application rates. However, even though N-emissions per ha increase under the intensification scenario (2), intensifying agriculture also leads to less area expansion which partially offsets the augmented total N-emission rates. The closed yield gaps scenario (4) results in lower N-emissions than the BAU path (1), because yield growth is assumed to be higher only for selected crops and total area growth is reduced. From a water quality perspective, this is good news, however total production volume is reduced due to the assumption of no increase in rainfed area. If this assumption were relaxed, N-emissions would very likely be higher under scenario 4 than under BAU (1). Very promising results are reflected in the sustainable intensification scenarios (3a) and (3b). While maintaining high yields, and thus production and trade volumes (with the positive effects for food security), the risk for water quality degradation would be much lower.

Under all scenarios, except for the sustainable intensification scenarios (3a) and (3b), there is an absolute increase in N-emissions between 2000 and 2050 in almost all important LAC’s agricultural production areas. (S4 Table ranks all FPUs according to their water degradation risk). Exceptions are those areas where yields are projected to decrease over time. Unsurprisingly in most FPUs, the steepest increase in N-emissions occur under the intensification scenario (2). Future hotspots of water degradation could be North-East Brazil (NEB_BRA), Amazon in Colombia (AMA_COL) and Coastal Peru (PEC_PER). However, if Precision Agriculture was applied (3b) in many FPUs the absolute amount of N-emissions would be reduced which would substantially reduce the risk for water degradation. For instance, in Middle Mexico (MIM_MEX) intensification (2) would increase N-emissions by almost 190% by 2050; however if Precision Agriculture was applied, total emissions could be almost 60% lower. This would not only mean a slower risk for degradation than under any other scenario, but a true improvement in 2050 compared to the base year 2000. One future hotspot with high risks for water pollution is the FPU North-South-America-Coast (NSA_NSA). The sharp increase in N-emissions is mainly caused by the N-emission increase on pasture land related to livestock production. The projected scale of excreta nitrogen produced in livestock production is relatively large with respect to the size of cropland and pasture land in this FPU, and therefore causes a sharp increase in N input rates on pasture land.

**Fig 5:**
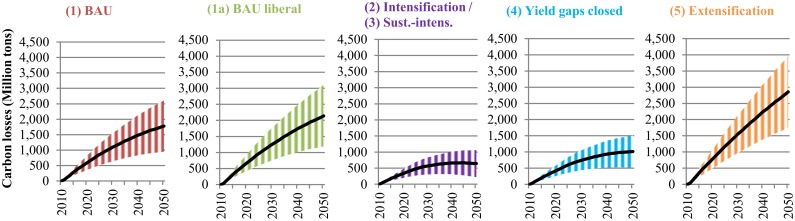
Annual net changes in carbon stock losses due to crop production under different scenarios in Latin America and the Caribbean (2010–2050). The values represent carbon stock losses from additional land conversion occurring in each year between 2010 and 2050. The shaded area illustrates carbon storage losses between a defined lower and upper bound due to different land expansion pathways. The lower bound reflects carbon storage losses if 100% of crop land expands over existing pasture land, while the upper bound reflects carbon storage losses if 100% of crop land expands over natural vegetation. The line illustrates the mean of the lower bound and upper bound. BAU refers to the Business-as-Usual scenario. Scenarios are described in Table 1. The intensification (2) and sustainable intensification (3) scenarios are presented together, because both scenarios have the same productivity assumptions and they only differ in terms of water consumption and nitrogen-emissions.

**Fig 6:**
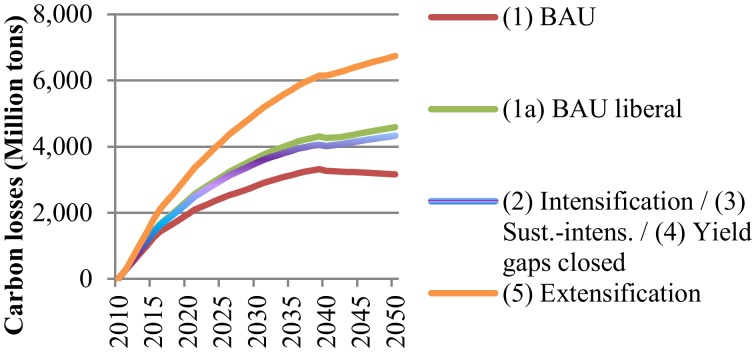
Annual net changes in carbon stock losses due to livestock production under different scenarios in Latin America and the Caribbean (2010–2050). The values represent carbon stock losses from additional land conversion occurring in each year between 2010 and 2050. BAU refers to the Business-as-Usual scenario. Scenarios are described in Table 1. The intensification (2) and sustainable intensification (3) scenarios are presented together, because both scenarios have the same productivity assumptions and they only differ in terms of water consumption and nitrogen-emissions.

**Impacts on carbon stock losses**. Fig. 5 illustrates changes in C stock losses from cropland expansion, while Fig. 6 shows C losses from future pasture land expansion due to livestock production in LAC. Significant net losses of C stocks are expected to occur in LAC by 2050, irrespectively of the scenario of agricultural development considered.

We find that C losses due to pasture land expansion from livestock production are even higher than C losses associated with crop production by 2050. This holds true, despite the fact that the conversion of natural land to pasture land releases less C than the conversion from natural land to croplands would do. As expected, the extensification scenario (5) would lead to the highest amount of carbon stock losses. Under this scenario approximately 106.7 million ha of natural land would be converted to pasture land. However, it is remarkable that the BAU scenario (1) shows lower C stock losses than the more intensive scenarios (2/3/4) which assume improved livestock yields. This finding points to the fact that further trade liberalization would strongly foster LAC’s comparative advantage in livestock production. This would in turn lead to expanding pasture land areas with the associated C stock losses. C stock losses from cropland expansion show a different picture. Here, the magnitude of total C stock losses do not only depend on the production scenario, but also on the land conversion pathway. If cropland spreads over natural vegetation, the C stock losses will be between 2.6 to 4.8 times larger compared to cropland expansion over existing pasture land. This can be explained by the fact that converting natural land releases much higher amounts of C than converting pasture land to cropland. Again, trade liberalization would augment C losses due to increased production of the seven crops for export markets (see scenario 1a). As expected, under the extensification scenario (5) the effect of trade liberalization would be exacerbated, because lower yields and higher rainfed area growth would lead to higher cropland expansion. Under this scenario, up to 53.6 million ha could be cleared for cultivation by 2050, implying a net change in carbon stocks between 1,747 and 3,966 million tons of C, depending on the land expansion pathway. The intensification scenarios (2/3/4) appear to be the paths with the lowest C footprint. Under these scenarios, new cultivated area in 2050 is expected to stay below 23 million ha, which would be equivalent to C stock losses of 222 to 1,511 million tons.

LAC features several hotspots of C losses due to crop production across FPUs in LAC (for details see S5 Table). Around 84% of the land cleared for crop cultivation is likely to occur in C-rich areas (FPUs with an average C content above 150 t/ha). Brazil and northern Argentina contribute the greatest carbon losses in absolute numbers due to substantial land conversion rates. The top FPUs experiencing C stock losses in Central America are Central America (CAM_CCA), Middle Mexico (MIM_MEX) and Yucatan (YUC_CCA). In the Central American FPU this can be attributed to high land conversion rates, while large losses in Yucatan are associated with substantial carbon storage in tropical forests. Hotspots of C losses from livestock production are different from those associated with cropland expansion (for details see S6 Table). In South America C losses will be highest in the Orinoco river basin in Northern-South-America (ORI_NSA), North-East Brazil (NEB_BRA), and the Amazon in Central-South America (AMA_CSA) and Peru (AMA_PER). In these areas pasture land increases from livestock production by 2050 will be between 42% and 47% under the BAU scenario (1) and between (57% and 90%) assuming extensification (5). In Central America highest C losses occur in the Caribbean (CAR_CCA) and Cuba (CUB_CCA). A few areas see gains in C stocks due to reduced cultivated and pasture areas by 2050, or because the new land is able to store more C than the original vegetation.

**Fig 7:**
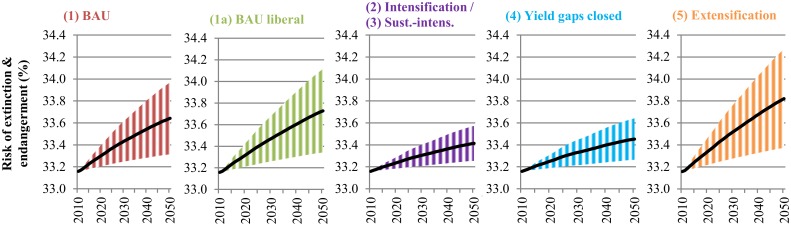
Annual species risk of extinction and endangerment due to crop production under different scenarios in Latin America and the Caribbean (2010–2050). The risk is expressed as an index in %. The shaded area illustrates the risk of biodiversity loss being between a defined lower and upper bound due to different land expansion pathways. The lower bound reflects the risk of biodiversity loss if 100% of crop land expands over existing pasture land, while the upper bound reflects the risk of biodiversity loss if 100% of crop land expands over natural vegetation. The line illustrates the mean of the lower bound and upper bound. BAU refers to the Business-as-Usual scenario. Scenarios are described in Table 1. The intensification (2) and sustainable intensification (3) scenarios are presented together, because both scenarios have the same productivity assumptions and they only differ in terms of water consumption and nitrogen-emissions.

**Impacts on biodiversity**. Fig. 7 shows that species’ risk of extinction and endangerment resulting from cropland expansion are likely to increase under all scenarios. However, the extent to which biodiversity will be at risk highly depends on the future production and land expansion pathway. Trade liberalization with its effects for agricultural production in LAC would increase the risk of biodiversity loss. Even more so, if yield growth rates slowed down and land growth rates accelerated as assumed under the extensification scenario (5). Intensifying production and closing yield gaps (scenarios 2/3/4) would lead to a much lower increase in species’ risk of extinction, even if the upper bound was considered. However, biodiversity impacts are overall less significant compared to C losses. Eighty-four percent of the projected increase in cropland under all scenarios is likely to be concentrated in a few FPUs, mostly those located in central, east and southern Brazil and northern Argentina. These FPUs contain 35% of the endemic bird richness of LAC. Yet, 60% of the Latin American birds’ endemicity is concentrated in FPUs where land clearing for crop cultivation will only make up 12% between 2010 and 2050, even if the extensification scenario (5) was considered. In these rich birds areas, current average species’ risk (*S*_*new*_/*S*_*org*_) is close to 34% which is likely to remain without major changes. Details on changes of biodiversity losses due to crop production across FPUs in LAC are given in S7 Table.

**Fig 8:**
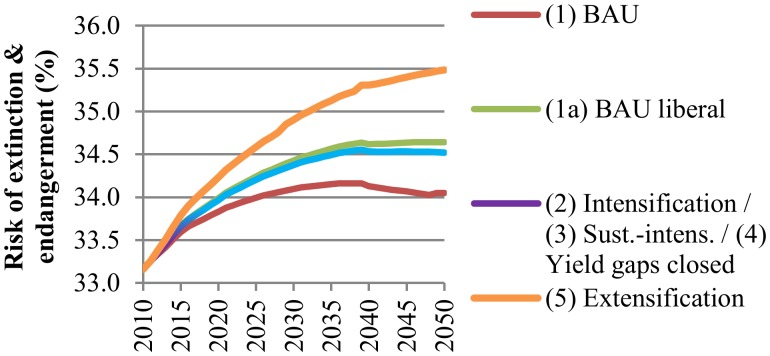
Annual species risk of extinction and endangerment due to livestock production under different scenarios in Latin America and the Caribbean (2010–2050). The risk is expressed as an index in %. BAU refers to the Business-as-Usual scenario.BAU refers to the Business-as-Usual scenario. Scenarios are described in Table 1. The intensification (2) and sustainable intensification (3) scenarios are presented together, because both scenarios have the same productivity assumptions and they only differ in terms of water consumption and nitrogen-emissions.

The overall increase in species’ risk of extinction and endangerment associated with pasture land expansion from livestock production will be higher than for cropland expansion (see Fig. 8). As with the carbon trade-offs, intensifying livestock production cannot offset the negative biodiversity effects from pasture land expansion resulting from trade liberalization. Biodiversity will be most affected by livestock production in FPUs located in South America, among them Tocantins in Brazil (TOC_BRA) or the Orinoco river basin in Northern-South-America (ORI_NSA). Especially the FPU Tocantins in Brazil already showed a relatively high risk of biodiversity loss in 2010. By 2050 more than half of the species will be critically endangered as a result of pasture land expansion. Although the risk of biodiversity loss in the Orinoco river basin was lower compared to Tocantins in 2010, by 2050 the share of species endangered will increase substantially due to livestock production. Under the BAU scenario (1) without trade liberalization risk will increase by 5.9 percentage points, while under the extensification scenario the risk will even increase by 10.5 percentage points. Details on risk of biodiversity losses due to livestock production across FPUs in LAC are provided in S8 Table.

## Discussion and Conclusion

Combining the analysis of agricultural trade with its various economic and environmental impacts has gained growing interest, especially for large exporting regions like LAC. This study presents an integrated approach assessing the effects of trade liberalization and different possible future production paths. We compare a global BAU scenario, which assumes a continuation of past trends in productivity improvements, area growth and technological change, with alternative future production paths developed for LAC. In terms of economic impacts, our model results are in line with the conclusions of other authors, e.g. [22, 56], showing that further trade liberalization leads to lower global prices for most crops and livestock products. Production, especially for export markets, shifts to those regions that hold comparative advantages in food production. Especially LAC’s livestock production would benefit from more open agricultural markets. This implies that trade liberalization is one way to improve global food security via higher global food supplies and lower prices. This does not mean, however, that there will not be regional winners and losers from trade policy reforms. In addition to agricultural trade, we find that different food production pathways will have differing effects on global food security.

On the basis of our partial equilibrium modeling, we find that extensifying agricultural production with low productivity improvements leads to lower production, net exports, and higher international food prices. An exception is soybean, where potential for productivity enhancement is lower, and the greatest opportunities for increased production are seen with rainfed land expansion, with clear trade-offs between land use and food production. However, land conversion is associated with substantial biodiversity losses and GHG emissions, a finding that is also confirmed by the literature, e.g. Fargione et al. (2008) [57]. We find these trade-offs will likely be concentrated in Brazil and northern Argentina. This points to the outstanding role of these two countries as global food—especially soybean—providers. The good news is that most of these FPUs are not among the biodiversity richest areas in LAC; but C stock losses would be considerable. The literature suggests that soybean expansion was the main driver of deforestation before 2005. But due to technical improvements and increased yields, followed by production expansion in previously cleared land, deforestation rates are now predominantly decoupled from soybean production. [10, 58]. Following this argument, we suggest to interpret our results of the environmental trade-offs in these Brazilian and Argentinian FPUs (see S5 and S7 Table) to be closer to the lower bounds of C stock and biodiversity losses, because new cropland will most likely not directly expand over natural vegetation. The literature also finds that bovine meat exports from South America continue to be correlated with the growth of permanent pastures, mostly at the expense of forests. [58, 59]. Our findings confirm that livestock production will lead to more natural land conversion than crop production in LAC by 2050, independent of the future production scenario. This holds true even under the assumption that only additional livestock farming will lead to an increase in pasture land by 2050. This will probably underestimate the reality though, because often crops expand over pasture land, which in turn crowds out livestock into forests. [58, 59]. Thus, the risks of biodiversity and C stock losses associated with livestock production should be interpreted as a lower bound in our study. Also, water resources will be affected, aggravating water scarcity and water pollution in some regions. Vast amounts of N fertilizers are used to grow farm animal feed, primarily composed of maize and soybeans [60]. So, if further global demand pressure leads to a continuation of past trends in soybean and livestock production, these countries will likely hit environmental limits with regional and global environmental consequences. Therefore, reducing meat consumption and thereby the demand for feedstuff as well as more responsible handling of food waste could be important ways to reducing demand pressures. Improvements in feeding efficiency (ratio of soybeans needed/kg meat produced) or promoting the consumption of meats with lower feed conversion losses (poultry) could make a contribution to reduce natural resource use. Even though it is difficult to change consumer habits, better information about healthy diets as well as environmental impacts of food consumption have shown some promise in the developed world [10].

Aside from soybean production, our results show that improving productivities would be the most effective way to ensure sufficient food production in the future. If yield growth were sufficiently high, even zero rainfed area growth could not offset higher production quantities and trade which would imply improved global food security. We should note though that a substantial fraction of LAC’ agricultural exports can be ascribed to energy crops (sugarcane). So increasing exports of these crops should have rather limited effects on improving global food security. Reconsidering biofuel policies in the developed world could create more area for food crops [61]. Our study also shows that more conventional intensification will come with environmental costs by further increasing water footprints and placing pressure on scarce water resources in some regions. Ercin and Hoekstra (2014) [23] come to similar results for water footprints in the future comparing different scenarios. Our results show that increasing basin efficiencies by improving irrigation technologies could offset the overuse of water to a certain extent. For instance, sub-surface drip irrigation technologies, coupled with modernized irrigation systems, have the potential to significantly increase water efficiencies. These techniques can be applied to crops like maize, sugarcane, alfalfa, cotton, and soybeans. Also, agriculture increasingly competes with other water users. Given continued growth in urbanization, the principles of cross-sectoral water resources management should offer strategies to harmonize competing uses and protect ecosystem services.

Moreover, conventional agricultural intensification would increase the risk of water quality degradation due to increased N-loadings. Water pollution could be offset by improving NUE or through the adoption of Precision Agriculture. These findings are in line with results from studies conducted by e.g. Liu et al. (2010) [62] and Bouwman et al. (2013) [63]. In the sustainable intensification scenario we assume an increase in NUE expressed as a fixed percentage rate of fertilizer input to crop output. This could be achieved through enhanced fertilizer use policies or breeding efforts. Precision Agriculture permits applying fertilizers where needs are most pressing, or where they generate the highest yield impacts. This however would mean large investments in new technologies, which might not be readily accessible by poorer farmers in those countries expecting the N pollution increases.

Results of the scenario in which yields gaps are closed show that closing yield gaps only in LAC is not sufficient to meet future global food demands, especially when combined with strict land conservation policies. The reason is that the discussed commercial crops already perform well in LAC compared to other world regions and therefore only a few areas in LAC show substantial yield gains. This does not mean that closing yield gaps is not valid to increase agricultural production to meet global food demand in 2050. For example, for many African countries, it will be important that yields catch up with other world regions [64]. Moreover, our results of potatoes in LAC show that closing yield gaps of this commodity will have strong positive market effects which could be environmentally friendly if natural resources were managed carefully.

Applying the global model IMPACT combined with environmental analyses yields many advantages, because it integrates inter-linked components, such as changes in climate, hydrology, water resources, or crop productivity. However, this is not without limitations. Due to the relatively coarse spatial resolution of the model, modeling water allocation at a more granular level is not possible, which might underestimate water scarcity in some areas of larger river basins. Furthermore, the hydrological model allows for groundwater pumping subject to an imposed capacity constraint, which might be an unrealistic assumption. Also, direct changes in livestock numbers currently do not feed back into the water supply and demand module, and are thus not reflected in scenarios of increased livestock numbers. In the extensification scenario, the BWF might therefore be underestimated. Finally, the agricultural research and development growth assumptions would imply close to immediate, significant investment. This is feasible, but agricultural research and development would have to move up on the priority scale in an increasingly urbanized society.

Apart from the limitations concerning the IMPACT model, there are other critical assumptions underlying our analysis. First of all, our alternative future scenarios only reflect production changes in LAC, while the rest of the world remains at BAU growth rates (except for trade liberalization which is assumed globally). Since our study looks at global food security indicators, it would likely be more meaningful if the improvements were applied at a global scale. However, our approach allows us to identify the specific role of a resource rich area like LAC for global food production. Another, more technical issue of our study is that the calculation of cultivated area is based on cropping intensities of the year 2000, approximately. This might lead to an overestimation of cultivated area with the corresponding environmental impacts in our study. Also, our pasture land estimations assume that live animals are based in the same FPUs as slaughtered animals, because IMPACT provides numbers of slaughtered animals. But animals are sometimes slaughtered in FPUs different from where they were raised. For example, according to Ramankutty et al. (2010) [65] some Central American FPUs show pasture areas in the base year, but without equivalent volumes of slaughtered animals in IMPACT. However, the key livestock producing FPUs in South America are well covered. Furthermore, we assume that the share of different livestock production systems (agro-pastoral, mixed extensive, mixed intensive) per FPU to remain constant at year 2000 levels. This will not fully reflect the reality, because LAC might further intensify livestock production in the future, a trend that will also be influenced by future climate change [6, 60]. Most importantly, our calculations are based on new pasture land from increasing livestock numbers only. This approach ignores possible dynamics between crop land expansion and displacements of pasture land from current livestock production [58, 59, 66]. In fact, pasture expansion, indirectly caused by farmland expansion and increasing exports of bovine meat, is considered the main cause of deforestation in the Brazilian Amazon [66]. Hence, our future pasture land estimates are likely to be too conservative. In parts, we approach these interdependencies between cropland and pasture land expansion by defining a lower and upper bound of future cropland expansion. However, there is a further source of complexity making it difficult to attribute direct causes of land use change. Reduced deforestation in one region can lead to land conversion in other regions. These highly dynamic processes make a precise modeling exercise in space extremely difficult, especially if the model operates at a large regional scale, like the IMPACT model. Efforts are therefore underway to integrate a land use model into IMPACT in order to capture these interdependencies.

Our study also intends to tackle environmental impacts of future reforestation in some regions in LAC. Aide et al. (2013) [67] found that between 2001 and 2010 LAC experienced intense deforestation (-541,835 *km* ^*2*^), but also reforestation (+362,430 *km* ^*2*^) processes. Forest transition (expansion and recovery of degraded forests) is common in Central America, Mexico, and in peri-urban ecosystems in South America, Andean forests and desserts and semi-arid ecosystems [4]. Since IMPACT results do not only provide increases in area harvested, but also decreases in some FPUs, we account for a possible recovery of natural vegetation. We assume that C stocks and biodiversity regain their original levels. However, the C stock losses and adverse biodiversity impacts may not be fully reversible. Grau & Aide (2008) [4] state that although marginal agricultural lands are being abandoned in many regions of Latin America, there is no guarantee that this will always lead to the recovery of natural ecosystems. On the one hand this might lead to an overestimation of the positive environmental impacts of reforestation in our study. On the other hand, a reduction in area harvested by 2050 is only estimated in a few FPUs in LAC, while the majority of FPUs show net increases.

In summary, we can state that Latin America is gaining in importance for supplying the rest of the world with food commodities. Due to market forces, LAC dedicates a large fraction of agricultural area to the production of livestock, feedstuff and biofuel crops. This means that wealthier population segments would benefit from the expansion of these products, rather than the poorest who consume staples. Nevertheless, limiting production by either insufficient yield increases or too strict land policies would not only reduce the production of soybeans and sugarcane, but also affect staple crops due to feedback effects between different agricultural commodities. So, increasing the amount of production is crucial (as are appropriate trade policies) for stabilizing world prices, and thereby improving food access of the poor. However, an increase of production comes at environmental costs in exporting nations. In order to reduce the environmental footprint of agricultural production without sacrificing future food security, policies must focus on promoting technological innovation that leads to higher yields without overusing water resources or polluting aquatic systems. Substantial yield increases could avoid excessive land use change with its devastating effects. Priority should be given to the adoption of existing sustainable technologies, good natural resource management inside and outside of agriculture, and most especially to investments in R&D in the agricultural sector (including, nutrients, pests, water and soils management, and improving plants’ performance in semi-arid conditions and salty soils). Furthermore, LAC could switch away from extensive livestock farming to feedlot systems, which would reduce pasture land expansion and associated adverse environmental consequences. Future research should be geared to identifying the economic and environmental impacts of global solutions, instead of focusing on specific regions.

## Supporting Information

S1 Text Sensitivity analysis to global climate models. (PDF)

S2 Text Projections of cropland and pasture land. (PDF)

S3 Text Water scarce Food Producing Units. (PDF)

S4 Text The IMPACT model. (PDF)

S5 Text Food security estimations. (PDF)

S6 Text Water footprint calculations. (PDF)

S7 Text Water quality assessment. (PDF)

S8 Text Assessment of carbon stock losses. (PDF)

S9 Text Biodiversity risk index assessment. (PDF)

S1 Fig Map of Food Producing Units in Latin America and the Caribbean. (TIF)

S2 Fig Validation of IMPACT area harvested projections for selected crops and large production regions in Latin America and the Caribbean from 2000 to 2012. IMPACT = International Model for Policy Analysis of Agricultural Commodities and Trade. The solid line (in blue) depicts IMPACT projections of area harvested and the dashed line (in red) represents data taken from FAOSTAT (2014). BAU refers to the Business-as-Usual scenario. MIROC climate change scenario assumptions were used which might differ slightly from other GCM results, because climate change is already assumed to show effects after year 2000. Although differences for this time span are rather low, MIROC’s predictions yield closer to reality results. IMPACT predicts the trends correctly, but understates changes in area harvested, so all results that refer to increases in agricultural area should be interpreted as conservative predictions.
(TIF)

S1 Table Definition of Food Producing Unit codes. (PDF)

S2 Table Food Producing Units with remaining yield gaps in 2050 in Latin America and the Caribbean (in tons/hectare). (PDF)

S3 Table Irrigation water supply reliability in selected water stressed Food Producing Units in Latin America and the Caribbean in 2010 and 2050. (PDF)

S4 Table Changes in nitrogen-emission rates between the base year 2000 and 2050 across different Food Producing Units in Latin America and the Caribbean (in %). (PDF)

S5 Table Net changes in carbon stock losses due to crop production across Food Producing Units in Latin America and the Caribbean between 2010 to 2050 (in million tons). (PDF)

S6 Table Net changes in carbon stock losses due to livestock production across Food Producing Units in Latin America and the Caribbean between 2010 to 2050 (in million tons). (PDF)

S7 Table Species risk of extinction and endangerment due to crop production across Food Producing Units in Latin America and the Caribbean in 2050 (index in %) and net changes between 2010 and 2050 (in %age points). (PDF)

S8 Table Species risk of extinction and endangerment due to livestock production across Food Producing Units in Latin America and the Caribbean in 2050 (index in %) and net changes between 2010 and 2050 (in %age points). (PDF)
