# Supplementary material for: The Role of Latin America’s Land and Water Resources for Global Food Security: Environmental Trade-Offs of Future Food Production Pathways
Source: PLoS One. 2015 Jan 24;10(1):e0116733. doi: 10.1371/journal.pone.0116733 (PMC4305321; doi:10.1371/journal.pone.0116733)
Supplement: S1 Text — (PDF) [file pone.0116733.s001.pdf]

S1 Text. Sensitivity analysis to global climate models

In the sensitivity analysis we analyze the same scenario specifications, but under different global climate models (GCM), namely CSIRO and MIROC. We focus on the outcomes of different climate change models, because different impacts of climate change reflect quite well existing uncertainties due to different future agricultural growing conditions. With identical GHG-emissions, the GCM climate outputs differ substantially. The CSIRO A1B scenario represents a dry and relatively cool future, while the MIROC A1B scenario represents a wet and warmer future at global scale. Such differences have yield effects and implications for irrigation water needs and in turn on land use patterns. In the main text all results presented stem from model runs using MIROC, because comparing reported FAO data between 2000 and 2010, MIROC seems to be closer to true values than model runs with CSIRO. On request the authors can provide all results presented in the paper also for the CSIRO CGM.

The results of the sensitivity analysis are summarized in Table A below. All numbers refer to the %-difference between the MIROC and CSIRO CGM in the year 2050, with MIROC being the reference model. The differences between the two climate change models in 2050 are relatively moderate, especially with respect to the aggregated land and water related variables. Deviations in aggregated area harvested and water footprints never exceed nine percent. The biggest deviations in area harvested appear in rice and maize production, but still being below 20 percent. Yield differences between the two GCMs are highest for maize, but do not pass 27 percent. The economic market effects are more pronounced, though. The largest deviation is observed for world prices of rice in 2050.

Table A. IMPACT estimation differences between MIROC and CSIRO CGMs in the year 2050 (in%)

Variable	Product	(1) BAU	(1a) BAU liberal	(2) Intensification	(3a/3b) Sustainable intensification	(4) Yield gaps closed	(5) Extensification
Green water footprint	All crops	-3%	-3%	-3%	-3%	-3%	-3%
Blue water footprint	All crops and livestock	-7%	-7%	-7%	-8%	-7%	-8%
Area harvested	All crops	-8%	-8%	-8%	-8%	-8%	-8%
Area harvested	Maize	-14%	-14%	-14%	-14%	-14%	-14%
Area harvested	Potatoes	-4%	-3%	-3%	-3%	-3%	-4%
Area harvested	Rice	-18%	-18%	-16%	-16%	-16%	-18%
Area harvested	Sorghum	0%	0%	0%	0%	1%	1%
Area harvested	Soybeans	-8%	-8%	-8%	-8%	-9%	-8%
Area harvested	Sugarcane	-3%	-3%	-3%	-3%	-3%	-3%
Area harvested	Wheat	-19%	-19%	-19%	-19%	-19%	-19%
Yield	Maize	24%	24%	27%	27%	24%	21%
Yield	Potatoes	14%	14%	16%	16%	13%	12%
Yield	Rice	-4%	-4%	-3%	-3%	-4%	-4%
Yield	Sorghum	4%	4%	5%	5%	4%	4%
Yield	Soybeans	3%	3%	4%	4%	2%	1%
Yield	Sugarcane	4%	4%	4%	4%	4%	3%
Yield	Wheat	13%	13%	12%	12%	13%	14%
World prices	Maize	-25%	-25%	-25%	-25%	-25%	-25%
World prices	Potatoes	-14%	-13%	-14%	-14%	-13%	-13%
World prices	Rice	-36%	-36%	-36%	-36%	-36%	-36%
World prices	Sorghum	-7%	-7%	-7%	-7%	-6%	-6%
World prices	Soybeans	-19%	-19%	-20%	-20%	-21%	-20%
World prices	Sugar	-12%	-12%	-12%	-12%	-11%	-11%
World prices	Wheat	-30%	-30%	-30%	-30%	-30%	-30%

Note: IMPACT = the International Model for Policy Analysis of Agricultural Commodities and Trade.
