# Supplementary material for: The Role of Latin America’s Land and Water Resources for Global Food Security: Environmental Trade-Offs of Future Food Production Pathways
Source: PLoS One. 2015 Jan 24;10(1):e0116733. doi: 10.1371/journal.pone.0116733 (PMC4305321; doi:10.1371/journal.pone.0116733)
Supplement: S2 Text — (PDF) [file pone.0116733.s002.pdf]

S2 Text. Projections of cropland and pasture land

We make a projection of the annual expansion of cropland and pasture areas between the year 2010 and 2050 under each agricultural production scenario to assess the carbon and biodiversity trade-offs over time. Furthermore, pasture land projections are also required for our water quality assessment which takes into account emissions from livestock production.

Cropland area

Model runs from the International Model for Policy Analysis of Agricultural Commodities and Trade (IMPACT) provide the projected annual area harvested per crop, Food Producing Unit (FPU) and scenario. Since the area harvested per crop might contain multiple harvests per year, annual cultivated area per FPU is calculated as:

$$Acrop_t = \sum_{i=1}^{7}\sum_{k=1}^{2}\frac{AH_{i,t,k}}{CI_{i,t,k}} \qquad \text{(Eq. S2.1)}$$

where $Acrop$ refers to the cultivated area of all seven crops (maize, rice, wheat, soybeans, sugarcane, potatoes, sorghum) in year t. CI is the cropping intensity of crop i and k accounts for rainfed and irrigated land. AH refers to annual area harvested of crop i under both, rainfed ($k = 1$) and irrigated conditions ($k = 2$). CI values are derived from You et al. (2013) [1].

Pasture area for livestock production

Projected pasture area here refers to the estimated land footprint required to sustain the expected increase in livestock production under the different scenarios. IMPACT does not directly provide results on annual pasture land requirements. Hence, we used the data on pasture land area by FPU provided by Ramankutty et al. (2010) [2] for the year 2000 as a reference point to make future pasture projections. Likewise, we converted the IMPACT outputs of projected head numbers of cows, sheep and goats (for pigs and chickens we assume that they are raised in landless/urban systems) by FPU into livestock units (LU), applying region-specific conversion factors provided by FAO (2011) [3]. Accordingly, projected annual pasture land by FPU is calculated as:

$$Apasture_t = \sum_{l=1}^{3} Apasture_{l,2000} * \frac{LU_{l,t}}{LU_{l,2000}} \qquad \text{(Eq. S2.2)}$$

where $Apasture_{2000}$ refers to the projected pasture area in year t and $Apasture_{l,2000}$ is the projected pasture area in the base year 2000 per livestock type l. $LU_{l,t}$ are the expected livestock units (heads per hectare) of type l in each future year t and $LU_{l,2000}$ refers to the livestock units of type l in the base year (2000).

Livestock densities may vary across different production systems. We assume the share of different livestock production systems (agro-pastoral, mixed extensive, mixed intensive) per FPU to remain constant at year 2000 levels. Hence, only changes in livestock numbers and livestock yields affect changes in the pasture land expansion, ignoring possible shifts towards more intensified livestock production systems, due to data limitations.

References

1. You L, Crespo S, Guo Z, Koo J, Ojo W, et al. (2013). Spatial Produciton Allocation Model (SPAM) 2000 Version 3 Release 2. URL http://mapspam.info/. Accessed 3 December 2013.

2. Ramankutty N, Evan A, Monfreda C, Foley J (2010). Global Agricultural Lands: Pastures, 2000. Palisades, NY: NASA Socioeconomic Data and Applications Center (SEDAC). URL http://dx.doi.org/10.7927/H47H1GGR. Accessed 15 October 2014.

3. FAO (2011) Guidelines for the preparation of livestock sector reviews. Technical report, Food and Agriculture Organization of the United Nations, Rome, Italy.
