# Supplementary material for: The Role of Latin America’s Land and Water Resources for Global Food Security: Environmental Trade-Offs of Future Food Production Pathways
Source: PLoS One. 2015 Jan 24;10(1):e0116733. doi: 10.1371/journal.pone.0116733 (PMC4305321; doi:10.1371/journal.pone.0116733)
Supplement: S3 Text — (PDF) [file pone.0116733.s003.pdf]

S3 Text. Water scarce Food Producing Units

Water scarcity is given where irrigation water supply reliability is smaller one or where total renewable water resources over total water withdrawals is larger than 20% at some point in time between 2010 and 2050. Food Producing Units (FPU) that suffer from water scarcity are:

- The Caribbean, Central-America (CAR_CCA)
- Cuba, Central-America (CUB_CCA)
- North-East Brazil (NEB_BRA)
- Parana, Brazil (PAR_BRA)
- Coastal Peru (PEC_PER)
- Salada-Tierra, Argentina (SAL_ARG)
- Tocantins, Brazil (TOC_BRA)
- Uruguay, Brazil (URU_BRA)
- Uruguay, Uruguay (URU_URU)
- Yucatan, Central-America (YUC_CCA)

A list of all FPUs can be found in S1 Table with the corresponding map in S1 Figure.
