# Supplementary material for: The Role of Latin America’s Land and Water Resources for Global Food Security: Environmental Trade-Offs of Future Food Production Pathways
Source: PLoS One. 2015 Jan 24;10(1):e0116733. doi: 10.1371/journal.pone.0116733 (PMC4305321; doi:10.1371/journal.pone.0116733)
Supplement: S4 Text — (PDF) [file pone.0116733.s004.pdf]

S4 Text. The IMPACT model

The International Model for Policy Analysis of Agricultural Commodities and Trade (IMPACT) uses supply and demand elasticities incorporated into a system of linear and nonlinear equations to represent the underlying production and demand functions for each product. World agricultural commodity prices are determined annually at levels that clear international markets. Domestic prices are a function of world prices, adjusted by the effects of price policies and expressed in terms of producer support estimates (PSE), consumer support estimates (CSE) and marketing margins (MI). PSEs and CSEs measure the implicit level of taxation or subsidy borne by producers or consumers relative to world prices and account for the wedge between domestic and world prices. PSEs and CSEs are based on OECD estimates and are adjusted by expert judgment to reflect regional trade dynamics [1]. MI reflect other factors such as transport and marketing costs of shipping goods to markets and are based on expert opinion on the quality and availability of transportation, communication, and market infrastructure. In our study, trade liberalization is implemented by adjusting these three parameters which affects producer and consumer prices. The following equations describe this relationship:

$$PS_{tni} = PW_i * (1 - MI_{tni}) * (1 + PSE_{tni}) \qquad \text{(Eq. S4.1)}$$

$$PD_{tni} = PW_i * (1 - MI_{tni}) * (1 - CSE_{tni}) \qquad \text{(Eq. S4.2)}$$

where PS = producer prices, PD = consumer prices, PW = world prices, MI = marketing margin, PSE = producer subsidy equivalent, CSE = producer subsidy equivalent, i = agricultural commodity, n = country and t = year. Since prices in turn affect food demand and supply, trade liberalization can be seen as one driver of changing global food security.

In addition to trade liberalization, we estimate the effects of different future production pathways on crop and livestock production in LAC. Growth in crop production in each Food producing Unit (FPU) is determined by adjusting the rates of area expansion and yield growth. We assume changes in the exogenous area growth rate to model higher or lower investments in irrigation. Also, land policies that that either allow for or restrict accelerated arable land growth are modeled by altering exogenous area growth rates. Adjustments in the exogenous yield growth rate capture different levels of investments in agricultural R&D, as well as yield responses to altered fertilization. Climate change impacts on yield and area growth are also captured in the exogenous growth rates. The corresponding model equations are as follows:

$$AC_{tni} = \alpha_{tni} * (PS_{tni})^{\varepsilon iin} * \prod_{j \neq i} (PS_{tnj})^{\varepsilon ijn} * (1 + gA_{tni} + \tau^{A}_{clim}) \qquad \text{(Eq. S4.3)}$$

$$YC_{tni} = \beta_{tni} * (PS_{tni})^{\gamma iin} * \prod_{k} (PF_{tnk})^{\gamma ikn} * (1 + gCY_{tni} + \tau^{y}_{clim}) \qquad \text{(Eq. S4.4)}$$

$$QS_{tni} = AC_{tni} * YC_{tni} \qquad \text{(Eq. S4.5)}$$

where AC = crop area, YC = crop yield, QS = quantity produced, PS = effective producer price, PF = price of inputs k (e.g. labor and fertilizer), i, j = commodity indices specific for crops, k = inputs such as labor and capital, n = country index, t = year, gA = exogenous growth rate of crop area, gCY = exogenous yield growth rate, τ^{A}_{clim} = area growth rate due to climate change, τ^{y}_{clim} = yield growth rate due to climate change, ε = area price elasticity, γ = yield price elasticity, α = crop area intercept, and β = crop yield intercept.

We also account for higher or lower degrees of intensification in the LACs' livestock sector. IMPACT models livestock production similarly to crop production except that livestock yield reflects only the effects of expected developments in technology which is the parameter we adjust in each scenario of our analysis. To change the total number of animals slaughtered, depending on the scenario assumptions, we adjust an exogenous trend variable. Besides the trend variable, the number of livestock slaughtered is a function of the livestock's own price and the price of competing commodities, as well as the prices of intermediate (feed) inputs. The following equations describe the livestock sector in IMPACT:

$$AL_{tni} = \alpha_{tni} * (PS_{tni})^{\varepsilon iin} * \prod_{j \neq i} (PS_{tnj})^{\varepsilon ijn} * \prod_{b \neq i} (PI_{tnb})^{\gamma ibn} * (1 + gSL_{tni}) \qquad \text{(Eq. S4.6)}$$

$$YL_{tni} = (1 + gLY_{tni}) * (YL_{t-1,ni}) \qquad \text{(Eq. S4.7)}$$

$$QS_{tni} = AL_{tni} * YL_{tni} \qquad \text{(Eq. S4.8)}$$

where AL = number of slaughtered livestock, YL = livestock product yield per head, PI = price of intermediate feed inputs, i, j = commodity indices specific for livstock, b = commodity index specific for feed crops, gSL = exogenous growth rate of number of slaughtered livestock, α = intercept of number of slaughtered livestock, ε = price elasticity of number of slaughtered livestock, and γ = feed price elasticity. The remaining variables are defined as for crop production.

Water is treated endogenously in IMPACT. Over time the water available for crop production varies due to changes in demographics, climate, and competing demand for water from other sectors of the economy. A certain share of available water in each river basin is first allocated to satisfy environmental flow requirements. Secondly, domestic water uses are satisfied which is highly influenced by the amount of people living in cities. An urban population usually uses more water than a rural population in absolute terms. In a third step, the IMPACT water model allocates water to industrial uses, in a fourth step to livestock and finally the remainder is allocated to crop production.

The effect of water stress on irrigated crop area comes from the DSSAT suite of crop models and analysis, using location specific information on climate, soils, and nitrogen application rates. Total irrigation water supply is allocated to crops according to crop water requirements incorporating changes in the hydrological cycle, including precipitation, runoff, and crop-specific potential evapotranspiration.

In IMPACT, the concept of basin efficiency is used to account for changes in irrigation efficiency within a river basin. It fully accounts for the portion of diverted irrigation water that returns to rivers or aquifer systems and can be reused repeatedly by downstream users. Basin efficiency is defined as the ratio of beneficial irrigation water consumption to total irrigation water consumption (TC) and effects beneficial consumption. In our sustainable intensification scenarios (5a) and (5b), we increase basin efficiencies by a certain factor which leads to higher beneficial water consumption. This is because irrigation water demand (see Eq. Eq. S4.9) is lower with higher basin efficiencies, and consequently a larger portion of demand is met compared to scenarios with lower efficiencies, therefore leading to higher beneficial water consumption.

$$IRWD = \frac{(NIRWD_i * AI_i) * (1 + LR)}{BE} \qquad \text{(Eq. S4.9)}$$

where $IRWD$ = total irrigation water demand, $NIRWD$ = net irrigation water demand (for details see Rosegrant (2012) [2]), AI" = Irrigated area, LR = Salt leaching factor, BE = Basin efficiency and i = commodity specific crop index.

References

1. OECD (2000). Producer and Consumer Support Estimates database. URL www.oecd.org/agriculture/pse. Accessed 2 Febuary 2014.

2. Rosegrant MW (2012) International Model for Policy Analysis of Agricultural Commodities and Trade (IMPACT): Model Description. Technical report, International Food Policy Research Institute (IFPRI), Washington D.C.
