# Supplementary material for: The Role of Latin America’s Land and Water Resources for Global Food Security: Environmental Trade-Offs of Future Food Production Pathways
Source: PLoS One. 2015 Jan 24;10(1):e0116733. doi: 10.1371/journal.pone.0116733 (PMC4305321; doi:10.1371/journal.pone.0116733)
Supplement: S5 Text — (PDF) [file pone.0116733.s005.pdf]

S5 Text. Food security estimations

Food security in different world regions is proxied by the total number of malnourished preschool children (under five years old). The cross-country regression suggested by Smith and Haddad (2000) [1] uses the following covariates: (1) the availability of calories, (2) clean water access, (4) female access to secondary education and female relative life-expectancy. By means of the estimated coefficients and base year and future values of each explanatory variable, we estimate the prevalence of malnourishment in different world regions. IMPACT simulations of each scenario used in our study provide base year data and future estimates on per capita calorie availability (covariate 1) by country. Base year data on the other covariates (2-4) are obtained from WDI (2010) [2], while future projections of the covariates (2-4) are taken from the United Nations Department of Economic and Social Affairs Population Division (2007) [3] and the Millennium Ecosystem Assessment (MEA) (2005) [4]. Note that, differences in future food security across our different scenarios owed to different IMPACT estimates of calorie availability in each country (covariate 1). The other covariates (2-4) change over time, but remain the same across scenarios.

References

1. Smith LC, Haddad LJ (2000) Explaining Child Malnutrition in Developing Countries: A Cross-country Analysis, volume 39. Washington D.C.: Intl Food Policy Research Institute (IFPRI), 112 pp.

2. WDI (2010). World Development Indicators. URL `http://data.worldbank.org`.

3. United Nations Department of Economic and Social Affairs Population Devision (2007) World Population Prospects: The 2006 Revision, Highlights. Technical report, Working Paper No. ESA/P/WP.202, New York.

4. MEA (2005) Millennium Ecosystem Assessment. Ecosystems and human well-being: synthesis. The Millennium Ecosystem Assessment series. Island Press, 155 pp.
