# Supplementary material for: The Role of Latin America’s Land and Water Resources for Global Food Security: Environmental Trade-Offs of Future Food Production Pathways
Source: PLoS One. 2015 Jan 24;10(1):e0116733. doi: 10.1371/journal.pone.0116733 (PMC4305321; doi:10.1371/journal.pone.0116733)
Supplement: S6 Text — (PDF) [file pone.0116733.s006.pdf]

S6 Text Water footprint calculations

IMPACT's water module calculates the annual GWF for each crop per FPU (in m^3/FPU) as follows (for simplicity of notation we refrain from indexing the FPU, year and scenario):

$$GWF_i = min \begin{cases} \sum_{m=1}^{M} ETM_{i,m} * AH_i, & \text{if } ETM_{i,m} < PE_{i,m} \\ \sum_{m=1}^{M} PE_{i,m} * AH_i, & \text{if } ETM_{i,m} \geq PE_{i,m} \end{cases} \quad \text{(Eq. S6.1)}$$

where $ETM_{i,m} = ET_{0_{i,m}} * K_{c_{i,m}}$. Furthermore, i = crop; m = month, with $M \leq 12$ (depending on cropping calendar); ETM = crop water depletion per unit crop; PE = effective precipitation per hectare; ET_0 = reference evapotranspiration; AH = area harvested and K_c = crop coefficient.

We define the annual blue water footprint of production (BWF) as total irrigation water (in m^3/FPU) applied per crop in each FPU which is calculated as follows:

$$BWF_i = min \begin{cases} \sum_{m=1}^{M} IRWD_{i,m}, & \text{if } IRWD_{i,m} \leq IRWS_{i,m} \\ \sum_{m=1}^{M} IRWS_{i,m}, & \text{if } IRWD_{i,m} > IRWS_{i,m} \end{cases} \quad \text{(Eq. S6.2)}$$

where $IRWD$ = total irrigation water demand and $IRWS$ = total irrigation water available for crop evapotranspiration. The model solves for total water that could be depleted in each month from surface and groundwater resources in each FPU, constrained by minimum environmental flow requirements and other water users' demand (domestic, industrial and livestock water uses). We can introduce future investments in new irrigation technologies and improved water management practices by improving the FPU's basin efficiency (for details see Text S1). A detailed methodology description on how the WSM calculates $IRWD$ and $IRWS$ can be found in Rosegrant (2012) [1].

References

1. Rosegrant MW (2012) International Model for Policy Analysis of Agricultural Commodities and Trade (IMPACT): Model Description. Technical report, International Food Policy Research Institute (IFPRI), Washington D.C.
