# Supplementary material for: The Role of Latin America’s Land and Water Resources for Global Food Security: Environmental Trade-Offs of Future Food Production Pathways
Source: PLoS One. 2015 Jan 24;10(1):e0116733. doi: 10.1371/journal.pone.0116733 (PMC4305321; doi:10.1371/journal.pone.0116733)
Supplement: S7 Text — (PDF) [file pone.0116733.s007.pdf]

S7 Text. Water quality assessment

The study assesses impacts of different production pathways on the water environement. In order to quantify the emissions of nitrogen-based pollutants related to each scenario, we apply the Soil and Water Assessment Tool (SWAT) [1] model and couple it to the International Model for Policy Analysis of Agricultural Commodities and Trade (IMPACT). For the base year SWAT simulation, grid-based estimates of farming area by crop, pasture land areas and livestock animal counts are obtained from Monfreda et al. (2008) [2], Ramankutty et al. (2008) [3] and Robinson et al. (2007) [4]. Data on crop and region specific fertilizer application rates for the year 2000 are taken from Mueller et al. (2012) [5]. Note that due to data limitations for year 2010, we had to take the base year 2000 for this analysis. In addition to fertilizer, manure also provides nitrogen input to agriculture land. Due to lacking data to track the fate of manure excreted from livestock production, we follow the approaches by Bouwman et al. (2009) [6] and Liu et al. (2010) [7], in which the total amounts of excreta accumulated in stables or on pasture land are calculated by multiplying the nitrogen excretion rates of livestock animals [8] by the size of livestock species. It is assumed that 90% of manure produced in stables are recycled to cropland [9] after an adjustment to account for the loss of NH_3 vitalization, as proposed by Bouwman et al. (1997) [10].

In order to analyze our future scenarios, we scale up gridded estimates of cropland areas, livestock animal counts and pasture land areas for the year 2000. Total future values of these variables aggregated to the Food Producing Unit (FPU) -level used in the SWAT simulation are matched to the values projected by IMPACT and the pasture land estimations at FPU-level (see Text S5) by assuming invariant spatial variability within each FPU. We also recalculate input rates of manure nitrogen to cropland and pasture land and estimate future fertilizer application rates for each of our future scenarios. We also estimate future fertilizer application rates and recalculate input rates of manure nitrogen to cropland and pasture land for each of our future scenarios. We project future fertilizer rates based on the increases in crop yields in each scenario, with a fertilizer yield response elasticity of 0.75. The elasticity is chosen following Valin et al. (2013) [11] who calculated the world average trend observed over the last 30 years. Results from IMPACT model runs provide information on yield changes for each of the seven crops in each FPU in LAC in 2050 which serve as the basis for calculating fertilizer application rates. Note that this procedure is not optimal for estimating future fertilizer needs, because of nonlinearities in the fertilizer-yield response. Similarly, we recalculate input rates of manure nitrogen on cropland and pasture land by assuming that the values of those key parameters used in the calculation, such as livestock animal nitrogen excretion rates and manure recycle rates to cropland, are unchanged rates over time. However, due to data limitations and the large regional scale of the paper, we need to stick to these rough estimations.

Finally, extra care is taken in the simulations for sustainable intensification. Under the sustainable intensification scenario with NUE improvement (3a), input rates of fertilizer and manure nitrogen on crop land are further adjusted according to calculated nitrogen input rates for the intensification scenario (2) and specified NUE enhancement (+20%). In the simulation for sustainable intensification with precision agriculture (3b), an auto-fertilization function in the SWAT model [12] is invoked to mimic the "intelligence" in fertilization operation in precision agriculture. The auto-fertilization function allows the model to determine the quantity and timing of nitrogen fertilizer/manure applications, given nitrogen requirements of different crop plants.

References

1. Arnold JG, Srinivasan R, Muttiah RS, Williams JR (1998) Large area hydrologic modeling and assessment part I: Model development. J Am Water Resour Assoc 34: 73–89.

2. Monfreda C, Ramankutty N, Foley JA (2008) Farming the planet: 2. Geographic distribution of crop areas, yields, physiological types, and net primary production in the year 2000. Global Biogeochem Cycles 22.

3. Ramankutty N, Evan AT, Monfreda C, Foley JA (2008) Farming the planet: 1. Geographic distribution of global agricultural lands in the year 2000. Global Biogeochem Cycles 22.

4. Robinson TP, Franceschini G, Wint W (2007) The Food and Agriculture Organization's Gridded Livestock of the World. Vet Ital 43: 745–51.

5. Mueller ND, Gerber JS, Johnston M, Ray DK, Ramankutty N, et al. (2012) Closing yield gaps through nutrient and water management. Nature 490: 254–257.

6. Bouwman AF, Beusen AHW, Billen G (2009) Human alteration of the global nitrogen and phosphorus soil balances for the period 1970-2050. Global Biogeochem Cycles 23: GB0A04.

7. Liu J, You L, Amini M, Obersteiner M, Herrero M, et al. (2010) A high-resolution assessment on global nitrogen flows in cropland. Proc Natl Acad Sci U S A 107: 8035–40.

8. Sheldrick W, Syers JK, Lingard J (2003) Contribution of livestock excreta to nutrient balances. Nutr Cycl Agroecosystems 66: 119–131.

9. Smil V (1999) Nitrogen in crop production: An account of global flows. Global Biogeochem Cycles 13: 647–662.

10. Bouwman AF, Lee DS, Asman WAH, Dentener FJ, Van Der Hoek KW, et al. (1997) A global high-resolution emission inventory for ammonia. Global Biogeochem Cycles 11: 561–587.

11. Valin H, Havlík P, Mosnier A, Herrero M, Schmid E, et al. (2013) Agricultural productivity and greenhouse gas emissions: trade-offs or synergies between mitigation and food security? Environ Res Lett 8 035019.

12. Neitsch S, Arnold J, Kiniry J, Williams J (2005) Soil and Water Assessment Tool Theoretical Documentation, Version 2005, Grassland. Technical report, Soil and Water Research Laboratory, USDA-ARS.
