# Supplementary material for: The Role of Latin America’s Land and Water Resources for Global Food Security: Environmental Trade-Offs of Future Food Production Pathways
Source: PLoS One. 2015 Jan 24;10(1):e0116733. doi: 10.1371/journal.pone.0116733 (PMC4305321; doi:10.1371/journal.pone.0116733)
Supplement: S8 Text — (PDF) [file pone.0116733.s008.pdf]

S8 Text. Assessment of carbon stock losses

Land use dynamics in LAC are highly complex. Since the International Model for Policy Analysis of Agricultural Commodities and Trade (IMPACT) is not a land use model, it cannot predict over which types of land uses crops and pastures are likely to expand over time. To assess the changes in carbon (C) stocks linked to cropland expansion under each scenario we assume two different land use pathways: (1) all new cropland area between 2010 and 2050 expands over existing pastures; and (2) new cropland area expansion takes place at the expense of former natural woody vegetation. Assessing the C trade-offs of both pathways, allows us to estimate a lower bound (*ClossLower*) and upper (*ClossUpper*) bound of C-losses associated with cropland expansion in Latin America and the Caribbean (LAC). For pastures, we assume that pasture area expansion related to livstock production between 2010 and 2050 occurs at the expense of former natural woody vegetation. Accordingly, annual losses in C stocks per Food Producing Unit (FPU) due to cropland or pasture land expansion are calculated as:

$$C_{lossUpper_t} = \sum_{i=1}^{7}(C_{biome} - C_{crop_i}) * \Delta Acrop_{i,t} \qquad \text{(Eq. S8.1)}$$

$$C_{lossLower_t} = \sum_{i=1}^{7}(C_{pasture} - C_{crop_i}) * \Delta Acrop_{i,t} \qquad \text{(Eq. S8.2)}$$

$$C_{lossPasture_t} = (C_{biome} - C_{pasture}) * \Delta Apasture_t \qquad \text{(Eq. S8.3)}$$

where C_{biome} is the average C content of natural woody vegetation, C_{crop} is the average content of C of crop i, and $C_{pasture}$ is the average content of carbon of pastures. ΔA_{crop} refers to the net area increase of crop i by FPU between the year 2010 and year t, and $\Delta A_{pasture}$ refers to the net area increase of pastures between 2010 and year t by FPU. C_{biome}, C_{crop}, and $C_{pastures}$ calculations include above- and belowground stocks.

Positive values of $C_{lossUpper_t}$, $C_{lossLower_t}$ and $C_{lossPasture_t}$ will imply a net loss of C stocks over time per FPU. Negative values will imply a net gain of C stocks which might occur under two circumstances: first, because new crop and/or pasture area are able to store higher carbon stocks than original land use; and secondly, because $\Delta Acrop_{i,t}$ or $\Delta Apasture_t$ could show a negative trend, implying a net reduction of crop and/or pasture area over time. In this case, we have assumed that abandoned agricultural crops and/or pastures are able to recover their maximum C storage capacity (C_{biome}). This allows us to account for the potential long-term impacts of agricultural abandonment and forest re-growth.

Aboveground C_{biome} and $C_{pasture}$ by FPU are calculated by summarizing the aboveground C stocks of natural woody vegetation and pastures from the 5 *arc minute* resolution (∼ 10 x 10 km) New IPCC Tier-1 Global Biomass Carbon Map [1]. Aboveground C_{crop} is calculated following the approach of West et al. (2010) [2] and is assumed to equal crop's net primary productivity (NPP_i), calculated as:

$$NPP_i = \frac{Y_i * DF_i * C}{HI_i * R_i} \qquad \text{(Eq. S8.4)}$$

where Y accounts for the average yield of a specific crop i and DF is the ratio of dry matter in crop i and was pre-defined to have a value of 0.85. C is a C content of 0.45 gr gr (C/gr) dry matter and considered to be equal for all crops. HI represents the harvested index (meaning the percent of biomass harvested that is used as food) and R is the proportion of belowground biomass with respect to total biomass. HI and R parameter values for each one of the seven crops were obtained from literature review (see e.g. [3, 4]) and are as follows:

Table B. Parameter values used to estimate NPP_i

Crop i	HI	R
Maize	0.52	0.18
Potatoes	0.55	0.10
Rice	0.50	0.09
Sorghum	0.52	0.09
Soybeans	0.42	0.15
Wheat	0.39	0.19
Sugarcane	0.85	0.35

Belowground C_{biome} is calculated by summarizing the 5 *arc minute* resolution ($\sim$ 10 x 10 km) global soil organic carbon map in the top 1 m [5] by FPU. We assume that conversion of natural vegetation and pastures to herbaceous croplands reduces soil carbon stocks by 50%. Likewise, the conversion of natural vegetation into pastures is assumed to reduce the soil carbon stock by 20%. These assumptions are consistent with Guo and Gifford (2002) [6] meta-analysis on soil carbon stocks variations due to land use changes.

References

1. Ruesch A, Gibbs H (2008). New IPCC Tier-1 Global Biomass Carbon Map For the Year 2000. Available online from the Carbon Dioxide Information Analysis Center, Oak Ridge National Laboratory, Oak Ridge, Tennessee. URL `http://cdiac.ornl.gov/. Accessed 27 Febuary 2014.`

2. West PC, Gibbs HK, Monfreda C, Wagner J, Barford CC, et al. (2010) Trading carbon for food: global comparison of carbon stocks vs. crop yields on agricultural land. Proc Natl Acad Sci U S A 107: 19645–19648.

3. Larcher W (2003) Physiological Plant Ecology: Ecophysiology and Stress Physiology of Functional Groups. Springer-Verlag Berlin Heidelberg New York, 513 pp.

4. Bradford JB, Lauenroth WK, Burke IC (2008) The impact of cropping on primary production in the U. S. Great Plains. Ecology 86: 1863–1872.

5. Hiederer R, Kochy N (2012) Global Soil Organic Carbon Estimates and the Harmonized World Soil Database. EUR 25225 EN. Ispra: Publications Office of the European Union, 79 pp.

6. Guo LB, Gifford RM (2002) Soil carbon stocks and land use change: A meta analysis. Glob Chang Biol 8: 345–360.
