# Supplementary material for: The Role of Latin America’s Land and Water Resources for Global Food Security: Environmental Trade-Offs of Future Food Production Pathways
Source: PLoS One. 2015 Jan 24;10(1):e0116733. doi: 10.1371/journal.pone.0116733 (PMC4305321; doi:10.1371/journal.pone.0116733)
Supplement: S9 Text — (PDF) [file pone.0116733.s009.pdf]

S9 Text. Biodiversity risk index assessment

We use the species-area relationship (SAR) to account for potential biodiversity trade-offs associated with each scenario of agricultural production in LAC. SAR models have been widely applied to account for biodiversity impacts linked to land use changes and habitat loss, e.g [1–4]. Specifically, we apply a countryside model [5] to predict endemic bird's risk of extinction and endangerment (S_{risk}) attributable to agricultural expansion. We limit the study to birds since taxon's sensitivity to different forms of land-use change is well studied, and data on their conservation status and spatial range are most reliable, updated, and readily available from IUCN (2008) [6] and CIESIN-Nature Server (2008) [7]. To avoid the scale dependency factor [1] when assessing the extinction rate we limit our study to endemic birds i.e., species with breeding range limited to Latin America and the Caribbean (LAC).

Original SAR models, also called conventional power models, assume that variations in species number is mainly a function of changes in habitat size. These models have been questioned as they tend to overestimate extinction rates [8, 9]. Overestimation is due to a large extend to the oversimplification of species-habitat relationships which ignore critical factors like differential responses of species to matrix composition, permeability and the existence of different degrees of habitat suitability, e.g. [10–12]. Countryside models partially fill some of the main gaps of the more conventional power models as they take into account taxon's affinity and adaptability to changing conditions, and can be formulated as:

$$S_{risk,t} = \frac{S_{new,t}}{S_{org}} = c\left(\frac{\sum_{j=1}^{4} h_j * A_{new,t}}{A_{org}}\right)^z \quad \text{(Eq. S9.1)}$$

where S_{new} is the number of bird species recorded in year t and S_{org} is the original number of bird species. A_{new} is the remaining habitat size in year t and $A_{org}$is the original habitat area. c is a constant and depends on the type of taxon and region, h_j represents the suitability of birds to habitat j, and z is also a constant indicating the rate of species change per unit of area [13]. Positive values of S_{risk} imply an increase in the birds' risk of extinction and endangerment, while negative values will entail a risk reduction over time. Negative values might occur as a result of a reduction in the agricultural area (cropland/pasture land) in some FPUs. To account for the impacts that agricultural abandonment and forest re-growth might have on biodiversity, we assume that h_j for these new (regrown) habitats equal those of natural vegetation. Information on the different habitat types j and $Anew$ by FPU in 2010 is obtained from the 300 meter resolution 2009 Global Land Cover Map [14]. Original land uses are grouped into four main classes (habitat types j): natural vegetation, cultivated area, cultivated pastures and urban/artificial. To determine the suitability of bird species to the different habitat types (h_j) we use the data provided by Koh and Ghazoul (2010) [2]. To improve model performance, h_j values are calibrated, and finally set to: $h = 1$ for natural vegetation; $h = 0.45$ for pastures; $h = 0.32$ for croplands and $h = 0$ for urban/artificial.

References

1. Brooks TM, Mittermeier RA, Mittermeier CG, Da Fonseca GAB, Rylands AB, et al. (2002) Habitat loss and extinction in the hotspots of biodiversity. Conserv Biol 16: 909–923.

2. Koh LP, Ghazoul J (2010) Spatially explicit scenario analysis for reconciling agricultural expansion, forest protection, and carbon conservation in Indonesia. Proc Natl Acad Sci U S A 107: 11140–11144.

3. Pimm SL, Askins RA (1995) Forest losses predict bird extinctions in eastern North America. Proc Natl Acad Sci U S A 92: 9343–9347.

4. Proenca V, Pereira HM (2013) Species-area models to assess biodiversity change in multi-habitat landscapes: The importance of species habitat affinity. Basic Appl Ecol 14: 102–114.

5. Pereira HM, Daily GC (2006) Modeling biodiversity dynamics in countryside landscapes. Ecology 87: 1877–1885.

6. IUCN (2008). International Union for Conservation of Nature, 2008 IUCN Red List of threatened species. IUCN, Gland, Switzerland. URL www.iucnredlist.org/. Accessed 13 March 2014.

7. CIESIN-Nature Server (2008). Center for International Earth Science Information Network (CIESIN)/Columbia University, and NatureServe, Gridded Species Distribution, Version 1: Birds of the Americas Presence Grids, Palisades, NY: NASA Socioeconomic Data and Applications Center (SEDAC). URL http://sedac.ciesin.columbia.edu/data/set/species-v1-americas-bird-presence. Accessed 5 March 2014.

8. He F, Hubbell SP (2011) Species-area relationships always overestimate extinction rates from habitat loss. Nature 473: 368–371.

9. Kinzig AP, Harte J (2000) Implications of endemics-area relationships for estimates of species extinctions. Ecology 81: 3305–3311.

10. Ricketts TH (2001) The matrix matters: effective isolation in fragmented landscapes. Am Nat 158: 87–99.

11. Revilla E, Wiegand T, Palomares F, Ferreras P, Delibes M (2004) Effects of matrix heterogeneity on animal dispersal: from individual behavior to metapopulation-level parameters. Am Nat 164: E130–E153.

12. Umetsu F, Paul Metzger J, Pardini R (2008) Importance of estimating matrix quality for modeling species distribution in complex tropical landscapes: A test with Atlantic forest small mammals. Ecography (Cop) 31: 359–370.

13. Koh LP, Ghazoul J (2010) A matrix-calibrated species-area model for predicting biodiversity losses due to land-use change. Conserv Biol 24: 994–1001.

14. ESA (2010). European Space Agency. Globcover 2009. Global Land Cover Map. URL due.esrin.esa.int/globcover/. Accessed 15 March 2014.
