# Supplementary figures and images for: The Role of Latin America’s Land and Water Resources for Global Food Security: Environmental Trade-Offs of Future Food Production Pathways
Source: PLoS One. 2015 Jan 24;10(1):e0116733. doi: 10.1371/journal.pone.0116733 (PMC4305321; doi:10.1371/journal.pone.0116733)

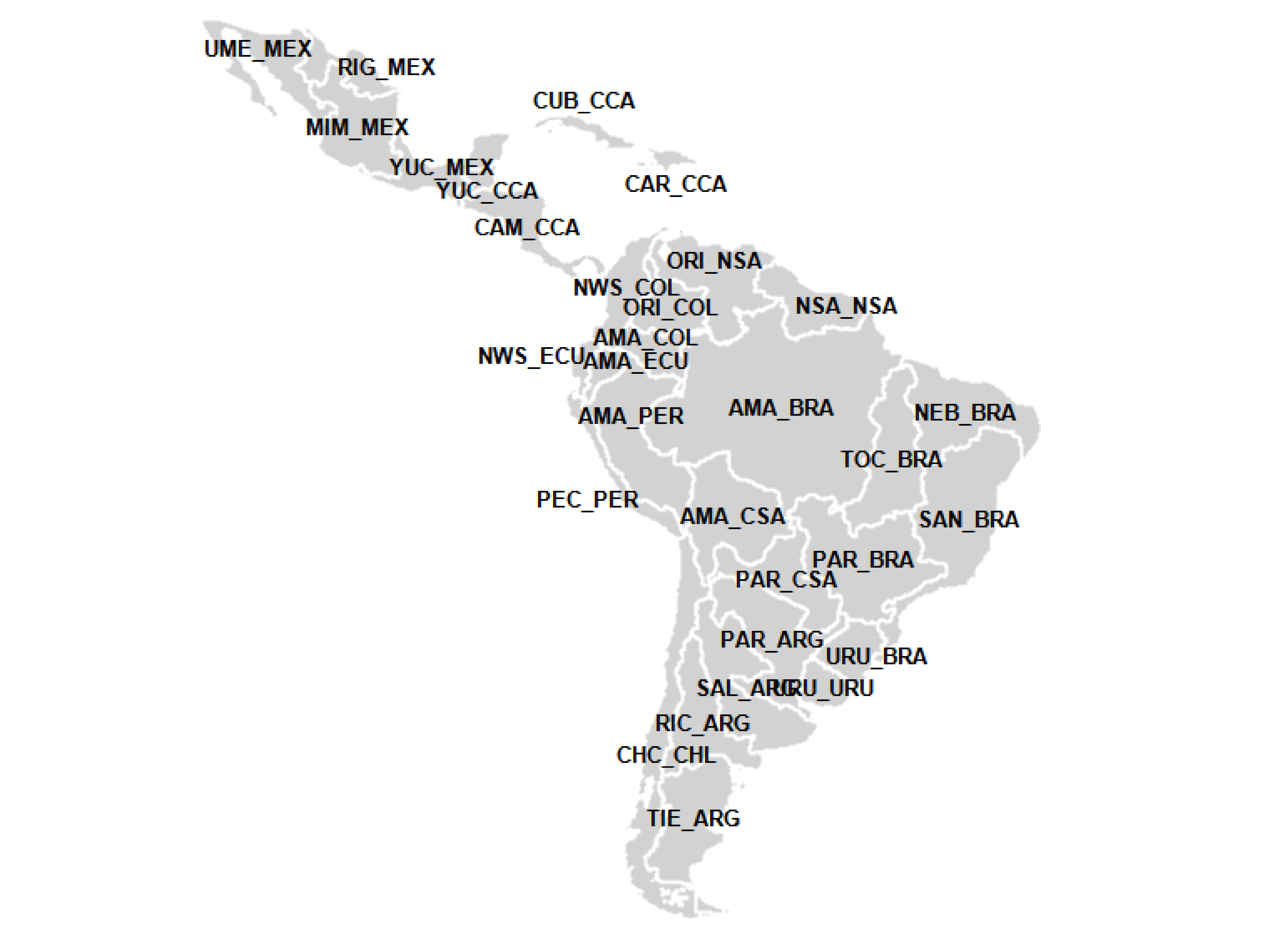

Supplement: S1 Fig — (TIF) [file pone.0116733.s010.tif]

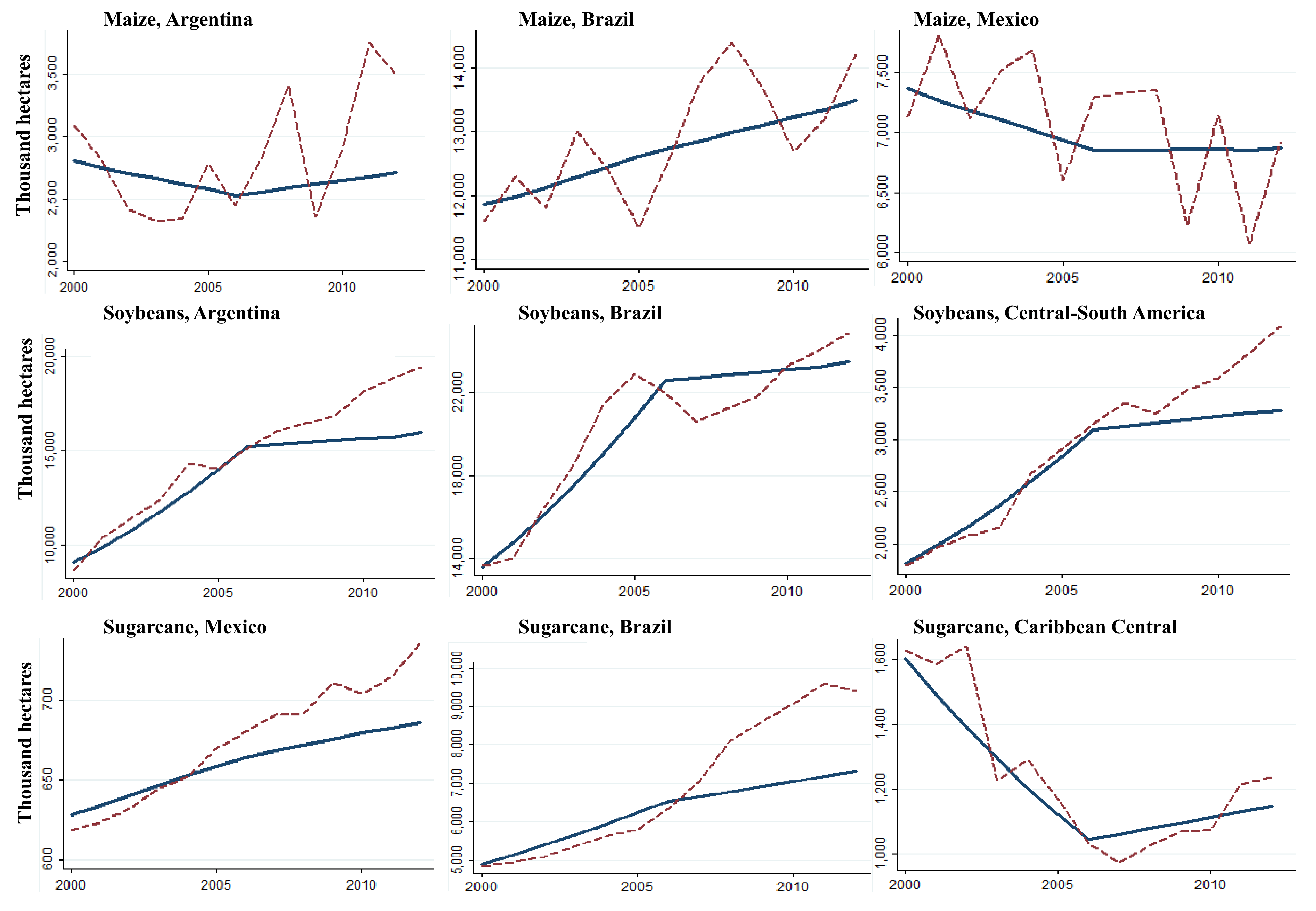

Supplement: S2 Fig — IMPACT = International Model for Policy Analysis of Agricultural Commodities and Trade. The solid line (in blue) depicts IMPACT projections of area harvested and the dashed line (in red) represents data taken from FAOSTAT (2014). BAU refers to the Business-as-Usual scenario. MIROC climate change scenario assumptions were used which might differ slightly from other GCM results, because climate change is already assumed to show effects after year 2000. Although differences for this time span are rather low, MIROC’s predictions yield closer to reality results. IMPACT predicts the trends correctly, but understates changes in area harvested, so all results that refer to increases in agricultural area should be interpreted as conservative predictions. (TIF) [file pone.0116733.s011.tif]
