# Supplementary material for: The Role of Latin America’s Land and Water Resources for Global Food Security: Environmental Trade-Offs of Future Food Production Pathways
Source: PLoS One. 2015 Jan 24;10(1):e0116733. doi: 10.1371/journal.pone.0116733 (PMC4305321; doi:10.1371/journal.pone.0116733)
Supplement: S1 Table — (PDF) [file pone.0116733.s012.pdf]

S1 Table. Definition of Food Producing Unit codes

FPU code	FPU in LAC
AMA_BRA	Amazon, Brazil
AMA_CSA	Amazon, Central-South-America
AMA_COL	Amazon, Colombia
AMA_ECU	Amazon, Ecuador
AMA_PER	Amazon, Peru
CAR_CCA	Caribbean, Caribbean-Central-America
CAM_CCA	Central-America, Caribbean-Central-America
CHC_CHL	Chile-Coast, Chile
CUB_CCA	Cuba, Caribbean-Central-America
MIM_MEX	Middle-Mexico, Mexico
NSA_NSA	North-South-America-Coast, Northern-South-America
NEB_BRA	Northeast-Brazil, Brazil
NWS_COL	Northwest-South-America, Colombia
NWS_ECU	Northwest-South-America, Ecuador
ORI_COL	Orinoco, Colombia
ORI_NSA	Orinoco, Northern-South-America
PAR_ARG	Parana, Argentina
PAR_BRA	Parana, Brazil
PAR_CSA	Parana, Central-South-America
PEC_PER	Peru-Coastal, Peru
RIC_ARG	Rio-Colorado, Argentina
RIG_MEX	Rio-Grande, Mexico
SAL_ARG	Salada-Tierra, Argentina
SAN_BRA	San-Francisco, Brazil
TIE_ARG	Tierra, Argentina
TOC_BRA	Toc, Brazil
UME_MEX	Upper Mexico, Mexico
URU_BRA	Uruguay, Brazil
URU_URU	Uruguay, Uruguay
YUC_CCA	Yucatan, Caribbean-Central-America
YUC_MEX	Yucatan, Mexico

Note: FPU = Food Producing Unit. To locate specific Food Producing Unit see S1 Figure. CCA = Belize, Costa Rica, Cuba, Dominican Republic, El Salvador, Guatemala, Haiti, Honduras, Nicaragua, Panama; CSA = Bolivia, Paraguay; NSA = Guyana, Surinam, Venezuela.
