# Supplementary material for: The Role of Latin America’s Land and Water Resources for Global Food Security: Environmental Trade-Offs of Future Food Production Pathways
Source: PLoS One. 2015 Jan 24;10(1):e0116733. doi: 10.1371/journal.pone.0116733 (PMC4305321; doi:10.1371/journal.pone.0116733)
Supplement: S2 Table — (PDF) [file pone.0116733.s013.pdf]

S2 Table. Food Producing Units with remaining yield gaps in 2050 in Latin America and the Caribbean (in tons/hectare)

FPU	Crop	Irrigated/ Rainfed	Observed yield, year 2000	Projected yield (BAU), year 2050	75% of attainable yield, year 2000	Yield gap, year 2000	Remaining yield gap, year 2050
**AMA_COL**	**Wheat**	**rainfed**	2.05	3.72	4.59	2.54	0.88
**AMA_CSA**	**Potatoes**	**irrigated**	11.62	18.87	26.30	14.68	7.43
**AMA_CSA**	**Potatoes**	**rainfed**	6.00	9.04	17.59	11.59	8.56
**AMA_ECU**	**Maize**	**irrigated**	2.03	5.59	6.09	4.06	0.50
**AMA_ECU**	**Maize**	**rainfed**	1.06	2.94	4.68	3.62	1.74
**AMA_ECU**	**Potatoes**	**irrigated**	9.01	13.92	26.08	17.06	12.16
**AMA_ECU**	**Potatoes**	**rainfed**	5.68	5.97	20.54	14.86	14.57
**AMA_ECU**	**Wheat**	**irrigated**	0.64	1.63	4.83	4.19	3.20
**AMA_PER**	**Maize**	**rainfed**	1.67	3.20	3.30	1.64	0.10
**AMA_PER**	**Potatoes**	**irrigated**	15.68	22.31	27.36	11.68	5.05
**AMA_PER**	**Potatoes**	**rainfed**	7.96	6.94	18.36	10.40	11.41
**AMA_PER**	**Soybeans**	**rainfed**	1.46	1.26	1.75	0.28	0.48
**AMA_PER**	**Wheat**	**rainfed**	1.28	2.02	4.09	2.81	2.07
**CAM_CCA**	**Maize**	**rainfed**	1.55	2.61	3.22	1.67	0.61
**CAR_CCA**	**Maize**	**rainfed**	1.54	2.17	2.81	1.27	0.64
**CAR_CCA**	**Sorghum**	**rainfed**	0.63	1.33	1.77	1.14	0.44
**CHC_CHL**	**Potatoes**	**rainfed**	14.36	14.17	22.79	8.42	8.61
**CUB_CCA**	**Maize**	**rainfed**	1.55	2.32	3.99	2.44	1.68
**CUB_CCA**	**Sorghum**	**rainfed**	0.64	1.18	1.95	1.32	0.77
**MIM_MEX**	**Maize**	**rainfed**	2.13	2.77	2.81	0.67	0.04
**MIM_MEX**	**Potatoes**	**rainfed**	13.89	14.40	16.90	3.01	2.51
**NSA_NSA**	**Maize**	**rainfed**	1.97	2.57	2.99	1.02	0.43
**NWS_COL**	**Maize**	**rainfed**	1.93	3.32	3.49	1.56	0.16
**NWS_ECU**	**Potatoes**	**irrigated**	9.03	13.95	21.67	12.64	7.72
**NWS_ECU**	**Potatoes**	**rainfed**	5.68	6.17	14.90	9.21	8.73
**NWS_ECU**	**Soybeans**	**rainfed**	1.80	1.31	1.89	0.09	0.58
**NWS_ECU**	**Wheat**	**irrigated**	0.65	1.67	3.34	2.69	1.67
**ORI_COL**	**Maize**	**rainfed**	1.93	3.33	3.75	1.82	0.42
**ORI_NSA**	**Wheat**	**rainfed**	0.39	1.50	1.60	1.22	0.11
**PAR_ARG**	**Potatoes**	**rainfed**	15.58	16.37	18.21	2.63	1.83
**PAR_CSA**	**Potatoes**	**irrigated**	11.58	18.71	27.19	15.61	8.48
**PAR_CSA**	**Potatoes**	**rainfed**	5.91	7.92	14.62	8.71	6.70
**PEC_PER**	**Maize**	**rainfed**	1.66	1.95	4.09	2.43	2.14
**PEC_PER**	**Potatoes**	**irrigated**	15.63	22.16	28.33	12.70	6.17
**PEC_PER**	**Potatoes**	**rainfed**	7.94	6.97	19.26	11.32	12.29
**PEC_PER**	**Wheat**	**rainfed**	1.27	1.41	2.31	1.04	0.90
**RIC_ARG**	**Potatoes**	**rainfed**	15.62	15.21	21.38	5.76	6.17
**RIG_MEX**	**Maize**	**rainfed**	2.14	2.62	4.33	2.19	1.71
**RIG_MEX**	**Potatoes**	**rainfed**	13.90	14.07	14.91	1.01	0.83
**SAL_ARG**	**Potatoes**	**rainfed**	15.66	16.19	17.97	2.30	1.77
**TIE_ARG**	**Potatoes**	**rainfed**	15.62	14.44	19.83	4.21	5.39
**UME_MEX**	**Maize**	**rainfed**	2.15	2.92	4.52	2.37	1.60
**URU_URU**	**Soybeans**	**rainfed**	1.79	1.64	2.15	0.36	0.51
**YUC_CCA**	**Maize**	**rainfed**	1.56	2.56	3.61	2.05	1.05
**YUC_CCA**	**Sorghum**	**rainfed**	0.64	1.55	2.21	1.57	0.66
**YUC_MEX**	**Wheat**	**rainfed**	3.17	3.02	3.40	0.23	0.39

Note: BAU refers to the Business-as-Usual scenario. Scenarios are described in Table 1 in the main text. FPU = Food Producing Unit. To locate Food Producing Units see S1 Figure and S1 Table.
