# Supplementary material for: The Role of Latin America’s Land and Water Resources for Global Food Security: Environmental Trade-Offs of Future Food Production Pathways
Source: PLoS One. 2015 Jan 24;10(1):e0116733. doi: 10.1371/journal.pone.0116733 (PMC4305321; doi:10.1371/journal.pone.0116733)
Supplement: S3 Table — (PDF) [file pone.0116733.s014.pdf]

S3 Table. Irrigation water supply reliability in selected water stressed Food Producing Units in Latin America and the Caribbean in 2010 and 2050

FPU	Year	(1) BAU	(1a) BAU liberal	(2) Intensification	(3) Sustainable intensification	(4) Yield gaps closed	(5) Extensification
**Central America and the Caribbean**							
**CUB_CCA**	**2010**	0.75	0.75	0.75	0.75	0.75	0.75
	**2050**	0.45	0.45	0.40	0.55	0.40	0.50
**YUC_CCA**	**2010**	0.57	0.57	0.57	0.57	0.57	0.57
	**2050**	0.70	0.69	0.68	0.92	0.68	0.70
**CAR_CCA**	**2010**	0.65	0.65	0.65	0.65	0.65	0.65
	**2050**	0.73	0.73	0.71	0.87	0.71	0.75
**South America**							
**TOC_BRA**	**2010**	0.11	0.11	0.11	0.11	0.11	0.11
	**2050**	0.10	0.11	0.11	0.11	0.11	0.10
**URU_BRA**	**2010**	0.21	0.21	0.21	0.21	0.21	0.21
	**2050**	0.41	0.42	0.40	0.54	0.40	0.44
**PEC_PER**	**2010**	0.67	0.67	0.67	0.67	0.67	0.67
	**2050**	0.44	0.44	0.42	0.57	0.42	0.45
**NEB_BRA**	**2010**	0.81	0.81	0.81	0.81	0.81	0.81
	**2050**	0.56	0.56	0.50	0.68	0.49	0.61
**SAL_ARG**	**2010**	0.96	0.96	0.96	0.96	0.96	0.96
	**2050**	0.73	0.72	0.66	0.88	0.65	0.78
**URU_URU**	**2010**	0.60	0.60	0.60	0.60	0.60	0.60
	**2050**	0.77	0.76	0.73	0.73	0.73	0.79
**PAR_BRA**	**2010**	1.00	1.00	1.00	1.00	1.00	1.00
	**2050**	0.83	0.82	0.72	0.97	0.70	0.93

Note: The indicator irrigation water supply reliability takes on values between zero and one, with lower values indicating severe water scarcity and values closer to one low water scarcity. FPU = Food Producing Unit. To locate Food Producing Units see S1 Figure and S1 Table. BAU refers to the Business-as-Usual scenario. Scenarios are described in Table 1 in the main text. Those FPUs are listed first that suffer the most from water stress under BAU (1) in 2050.
