# Supplementary material for: The Role of Latin America’s Land and Water Resources for Global Food Security: Environmental Trade-Offs of Future Food Production Pathways
Source: PLoS One. 2015 Jan 24;10(1):e0116733. doi: 10.1371/journal.pone.0116733 (PMC4305321; doi:10.1371/journal.pone.0116733)
Supplement: S4 Table — (PDF) [file pone.0116733.s015.pdf]

S4 Table. Changes in nitrogen-emission rates between the base year 2000 and 2050 across different Food Producing Units in Latin America and the Caribbean (in %)

FPU	(1) BAU	(1a) BAU liberal	(1.6) Intensification	(3a) Sustainable intensification (Improved NUE)	(3b) Sustainable intensification (Precision agriculture)	(4) Yield gaps closed	(5) Extensification
**Central America and the Caribbean**							
**MIM_MEX**	197	208	186	134	-59	238	197
**YUC_MEX**	97	104	99	75	-14	106	87
**CAR_CCA**	79	86	94	66	-6	91	82
**CAM_CCA**	41	45	41	31	-39	51	43
**YUC_CCA**	16	19	8	16	-20	33	35
**UME_MEX**	-34	-34	-38	-38	-53	-29	-32
**CUB_CCA**	-39	-37	-41	-45	-70	-39	-44
**RIG_MEX**	-53	-53	-55	-55	-62	-51	-52
**South America**							
**NSA_NSA**	1164	1239	1432	1202	1156	1211	1209
**NEB_BRA**	444	453	488	384	293	415	409
**AMA_COL**	381	399	434	392	365	398	393
**PEC_PER**	249	255	246	256	226	264	262
**URU_URU**	225	257	312	245	209	254	252
**AMA_ECU**	197	219	258	199	158	212	223
**SAL_ARG**	129	141	154	89	63	104	102
**ORI_NSA**	121	139	157	127	58	148	135
**PAR_ARG**	117	128	143	83	70	90	92
**CHC_CHL**	108	136	120	100	-44	163	138
**NWS_ECU**	99	108	107	75	-44	109	97
**PAR_BRA**	89	98	100	63	-11	82	73
**PAR_CSA**	83	91	107	84	74	87	88
**AMA_CSA**	76	92	117	83	70	86	87
**AMA_PER**	70	83	95	67	13	86	86
**URU_BRA**	63	68	72	46	37	49	50
**NWS_COL**	56	69	73	58	25	66	65
**AMA_BRA**	56	66	72	42	14	50	47
**ORI_COL**	55	55	55	53	42	55	54
**SAN_BRA**	51	59	63	32	-24	47	41
**TOC_BRA**	27	29	37	19	13	21	19
**TIE_ARG**	-6	-6	-6	-6	-6	-6	-6
**RIC_ARG**	-12	-11	-14	-13	-26	-9	-11

FPU = Food Producing Unit. To locate Food Producing Units see S1 Figure and S1 Table. Note: The table is organized in such a way that those Food Producing Units (FPU) where the increase in N-emissions are highest under (1) BAU, appear in the upper rows. BAU refers to the Business-as-Usual scenario. Scenarios are described in Table 1 in the main text.
