# Supplementary material for: The Role of Latin America’s Land and Water Resources for Global Food Security: Environmental Trade-Offs of Future Food Production Pathways
Source: PLoS One. 2015 Jan 24;10(1):e0116733. doi: 10.1371/journal.pone.0116733 (PMC4305321; doi:10.1371/journal.pone.0116733)
Supplement: S5 Table — (PDF) [file pone.0116733.s016.pdf]

S5 Table. Net changes in carbon stock losses due to crop production across Food Producing Units in Latin America and the Caribbean between 2010 to 2050 (in million tons)

	(1) BAU		(1a) BAU liberal		(2) Intensification / (3) Sust.-intens.		(4) Yield gaps closed		(5) Extensification	
**FPU**	**lower bound**	**upper bound**	**lower bound**	**upper bound**	**lower bound**	**upper bound**	**lower bound**	**upper bound**	**lower bound**	**upper bound**
**Central America and the Caribbean**										
CAM_CCA	22	58	28	67	5	22	12	33	40	86
MIM_MEX	7	20	9	24	-20	-31	-16	-22	20	42
YUC_CCA	8	18	9	20	4	8	5	10	12	25
CUB_CCA	-25	7	-26	12	-32	11	-22	12	-14	12
CAR_CCA	-1	4	-1	4	-4	-2	-2	1	2	7
YUC_MEX	1	2	3	6	-20	-40	-19	-36	10	19
RIG_MEX	-1	-1	-1	-1	-2	-3	-1	-2	0	-1
UME_MEX	-2	-3	-2	-2	-5	-9	-4	-7	-1	-1
**South America**										
PAR_ARG	336	691	375	773	151	328	183	385	452	912
PAR_BRA	25	394	58	476	-62	133	21	250	219	707
SAL_ARG	126	329	141	368	48	159	70	194	183	442
AMA_BRA	127	241	145	276	40	82	60	115	188	347
URU_BRA	132	214	149	242	67	114	87	141	183	291
SAN_BRA	24	207	51	267	4	115	35	161	107	356
TOC_BRA	57	132	65	149	14	42	27	64	92	194
NEB_BRA	47	121	75	167	9	46	29	73	116	225
PAR_CSA	31	56	43	75	22	42	32	56	54	93
ORI_NSA	8	45	9	49	2	31	6	39	13	53
AMA_CSA	13	31	19	44	8	19	11	25	24	53
AMA_PER	9	31	12	41	7	28	8	30	14	43
NWS_COL	4	10	8	18	-1	1	1	4	12	23
AMA_ECU	4	8	5	9	4	7	4	7	5	9
URU_URU	3	4	5	8	3	4	3	5	6	9
ORI_COL	0	2	1	4	-1	-3	-1	-2	1	6
NWS_ECU	2	1	4	4	-1	-3	-1	-3	4	5
NSA_NSA	0	1	1	2	-1	-2	-1	-2	1	3
AMA_COL	0	1	1	2	0	0	0	0	1	3
TIE_ARG	0	0	0	0	0	0	0	0	0	0
PEC_PER	-4	-4	-4	-3	-8	-7	-6	-5	-2	-1
RIC_ARG	-6	-4	-6	-4	-11	-8	-8	-6	-4	-2
CHC_CHL	3	-6	6	-6	4	-20	6	-10	6	7

Note: The values represent carbon stock losses from additional land conversion between 2010 and 2050. Positive values should be interpreted as a loss in carbon stock, and thus higher carbon emissions. The lower bound reflects carbon storage losses if 100% of crop land expands over existing pasture land, while the upper bound reflects carbon storage losses if 100% of crop land expands over natural vegetation. Considered crops in the analysis are maize, rice, wheat, soybeans, sugarcane, potatoes, sorghum. FPU = Food Producing Unit. To locate Food Producing Units see S1 Figure and S1 Table. BAU refers to the Business-as-Usual scenario. Scenarios are described in Table 1 in the main text. Those FPUs are listed first that show the highest total losses of carbon stocks according to the upper bound land expansion pathway under the BAU scenario.
