# Supplementary material for: The Role of Latin America’s Land and Water Resources for Global Food Security: Environmental Trade-Offs of Future Food Production Pathways
Source: PLoS One. 2015 Jan 24;10(1):e0116733. doi: 10.1371/journal.pone.0116733 (PMC4305321; doi:10.1371/journal.pone.0116733)
Supplement: S6 Table — (PDF) [file pone.0116733.s017.pdf]

S6 Table. Net changes in carbon stock losses due to livestock production across Food Producing Units in Latin America and the Caribbean between 2010 to 2050 (in million tons)

FPU	(1) BAU	(1a) BAU liberal	(2) Intensification / (3) Sust.-intens.	(4) Yield gaps closed	(5) Extensification
**Central America and the Caribbean**					
CAR_CCA	39	65	61	61	94
CUB_CCA	20	63	57	57	111
YUC_MEX	43	59	54	53	94
MIM_MEX	18	25	23	23	40
CAM_CCA	0	0	0	0	0
RIG_MEX	0	0	0	0	0
UME_MEX	0	0	0	0	0
YUC_CCA	0	0	0	0	0
**South America**					
ORI_NSA	803	1081	1032	1033	1534
NEB_BRA	627	629	625	626	852
TOC_BRA	603	606	601	602	820
AMA_CSA	344	468	448	448	663
AMA_PER	254	340	327	326	480
URU_URU	254	330	316	316	475
PAR_CSA	220	259	255	253	361
PAR_ARG	132	223	188	186	332
SAL_ARG	69	128	105	103	193
AMA_ECU	78	100	95	95	142
AMA_BRA	-482	73	29	25	184
NSA_NSA	54	73	69	69	103
AMA_COL	59	60	58	58	82
NWS_ECU	43	50	49	49	69
CHC_CHL	0	0	0	0	0
ORI_COL	0	0	0	0	0
PEC_PER	0	0	0	0	0
RIC_ARG	0	0	0	0	0
TIE_ARG	0	0	0	0	0
URU_BRA	0	0	0	0	0
NWS_COL	-19	-3	-4	-5	22
SAN_BRA	-65	-19	-27	-28	68
PAR_BRA	-64	-21	-29	-30	15

Note: The values represent carbon stock losses from additional land conversion between 2010 and 2050. Positive values should be interpreted as a loss in carbon stock, and thus higher carbon emissions. It is assumed that pasture land due to increasing livestock production entirely expands over natural vegetation. FPU = Food Producing Unit. To locate Food Producing Units see S1 Figure and S1 Table. BAU refers to the Business-as-Usual scenario. Scenarios are described in Table 1 in the main text. Those FPUs are listed first that show the highest total losses of carbon stocks under the BAU scenario.
