# Supplementary material for: The Role of Latin America’s Land and Water Resources for Global Food Security: Environmental Trade-Offs of Future Food Production Pathways
Source: PLoS One. 2015 Jan 24;10(1):e0116733. doi: 10.1371/journal.pone.0116733 (PMC4305321; doi:10.1371/journal.pone.0116733)
Supplement: S7 Table — (PDF) [file pone.0116733.s018.pdf]

S7 Table. Species risk of extinction and endangerment due to crop production across Food Producing Units in Latin America and the Caribbean in 2050 (index in %) and net changes between 2010 and 2050 (in %age points)

	(1) BAU		(1a) BAU liberal		(2) Intensification / (3) Sust.-intens.		(4) Yield gaps closed		(5) Extensification	
**FPU**	**lower bound**	**upper bound**	**lower bound**	**upper bound**	**lower bound**	**upper bound**	**lower bound**	**upper bound**	**lower bound**	**upper bound**
**Central America and the Caribbean**										
CAM_CCA	44.4 (+0.1)	44.9 (+0.6)	44.4 (+0.1)	45.0 (+0.7)	44.3 (+0.0)	44.5 (+0.2)	44.4 (+0.1)	44.6 (+0.3)	44.5 (+0.2)	45.2 (+0.9)
CAR_CCA	57.8 (+0.1)	58.2 (+0.6)	57.8 (+0.1)	58.3 (+0.6)	57.7 (+0.1)	58.0 (+0.3)	57.7 (+0.1)	58.0 (+0.4)	57.8 (+0.1)	58.3 (+0.6)
CUB_CCA	25.2 (+0.0)	25.3 (+0.1)	25.3 (+0.0)	25.4 (+0.2)	25.3 (+0.0)	25.4 (+0.2)	25.3 (+0.0)	25.4 (+0.2)	25.2 (+0.0)	25.4 (+0.2)
MIM_MEX	28.9 (+0.0)	28.9 (+0.0)	29.0 (+0.0)	29.0 (+0.1)	28.8 (-0.2)	28.1 (-0.8)	28.8 (-0.1)	28.3 (-0.6)	29.0 (+0.1)	29.2 (+0.3)
RIG_MEX	28.5 (+0.0)	28.5 (+0.0)	28.5 (+0.0)	28.5 (+0.0)	28.5 (+0.0)	28.4 (-0.1)	28.5 (+0.0)	28.4 (-0.1)	28.5 (+0.0)	28.5 (+0.0)
UME_MEX	28.6 (+0.0)	28.4 (-0.2)	28.6 (+0.0)	28.4 (-0.2)	28.6 (-0.1)	28.4 (-0.3)	28.6 (-0.1)	28.4 (-0.3)	28.6 (+0.0)	28.5 (-0.2)
YUC_MEX	33.5 (+0.0)	33.3 (-0.2)	33.5 (+0.0)	33.4 (-0.2)	33.4 (-0.2)	32.6 (-0.9)	33.4 (-0.2)	32.7 (-0.8)	33.6 (+0.0)	33.6 (+0.0)
**South America**										
PAR_ARG	47.0 (+0.7)	49.7 (+3.4)	47.1 (+0.8)	50.2 (+3.8)	46.7 (+0.3)	47.9 (+1.5)	46.7 (+0.3)	48.1 (+1.7)	47.3 (+0.9)	50.8 (+4.4)
PAR_BRA	58.1 (+0.6)	60.6 (+3.0)	58.2 (+0.7)	61.0 (+3.5)	57.8 (+0.3)	59.0 (+1.4)	57.9 (+0.3)	59.2 (+1.7)	58.3 (+0.8)	61.6 (+4.1)
SAL_ARG	49.1 (+0.5)	51.3 (+2.7)	49.1 (+0.6)	51.6 (+3.1)	48.8 (+0.3)	50.0 (+1.5)	48.8 (+0.3)	50.2 (+1.6)	49.2 (+0.7)	52.0 (+3.5)
CHC_CHL	41.9 (+0.5)	44.0 (+2.6)	42.0 (+0.5)	44.1 (+2.7)	41.7 (+0.3)	43.0 (+1.5)	41.7 (+0.3)	43.1 (+1.6)	42.0 (+0.5)	44.2 (+2.7)
URU_BRA	58.9 (+0.5)	60.8 (+2.4)	59.0 (+0.6)	61.3 (+2.8)	58.7 (+0.2)	59.6 (+1.2)	58.7 (+0.3)	59.8 (+1.4)	59.1 (+0.6)	61.7 (+3.3)
SAN_BRA	52.9 (+0.2)	53.9 (+1.3)	52.9 (+0.3)	54.1 (+1.5)	52.8 (+0.1)	53.3 (+0.7)	52.8 (+0.2)	53.4 (+0.8)	53.0 (+0.3)	54.4 (+1.8)
NEB_BRA	48.8 (+0.2)	49.5 (+0.9)	48.9 (+0.2)	49.8 (+1.2)	48.7 (+0.1)	49.0 (+0.4)	48.7 (+0.1)	49.1 (+0.5)	48.9 (+0.3)	50.0 (+1.4)
TOC_BRA	45.9 (+0.2)	46.6 (+0.8)	45.9 (+0.2)	46.7 (+0.9)	45.8 (+0.0)	45.9 (+0.2)	45.8 (+0.1)	46.0 (+0.3)	46.0 (+0.2)	46.9 (+1.2)
AMA_BRA	20.2 (+0.1)	20.5 (+0.5)	20.2 (+0.1)	20.6 (+0.5)	20.1 (+0.0)	20.2 (+0.1)	20.1 (+0.0)	20.2 (+0.2)	20.2 (+0.1)	20.7 (+0.7)
PAR_CSA	37.5 (+0.1)	37.8 (+0.4)	37.5 (+0.1)	38.1 (+0.6)	37.5 (+0.1)	37.7 (+0.3)	37.5 (+0.1)	37.8 (+0.4)	37.6 (+0.2)	38.2 (+0.8)
ORI_NSA	35.7 (+0.1)	36.0 (+0.3)	35.7 (+0.1)	36.0 (+0.4)	35.7 (+0.0)	35.9 (+0.3)	35.7 (+0.1)	36.0 (+0.3)	35.7 (+0.1)	36.0 (+0.3)
AMA_ECU	27.4 (+0.1)	27.7 (+0.3)	27.4 (+0.1)	27.7 (+0.3)	27.4 (+0.0)	27.6 (+0.3)	27.4 (+0.1)	27.6 (+0.3)	27.4 (+0.1)	27.7 (+0.4)
RIC_ARG	16.2 (+0.1)	16.4 (+0.3)	16.2 (+0.1)	16.4 (+0.3)	16.2 (+0.1)	16.4 (+0.3)	16.2 (+0.1)	16.5 (+0.3)	16.2 (+0.0)	16.4 (+0.3)
PEC_PER	20.5 (+0.0)	20.7 (+0.2)	20.6 (+0.1)	20.9 (+0.4)	20.6 (+0.1)	20.9 (+0.4)	20.6 (+0.1)	20.9 (+0.4)	20.5 (+0.1)	20.7 (+0.3)
AMA_PER	18.9 (+0.0)	19.0 (+0.2)	18.9 (+0.1)	19.1 (+0.3)	18.9 (+0.0)	19.0 (+0.2)	18.9 (+0.0)	19.0 (+0.2)	18.9 (+0.1)	19.1 (+0.3)
AMA_CSA	26.7 (+0.0)	26.9 (+0.2)	26.8 (+0.1)	27.0 (+0.3)	26.7 (+0.0)	26.8 (+0.1)	26.7 (+0.0)	26.8 (+0.1)	26.8 (+0.1)	27.0 (+0.3)
NWS_COL	41.7 (+0.0)	41.8 (+0.1)	41.7 (+0.0)	41.9 (+0.2)	41.7 (+0.0)	41.7 (+0.0)	41.7 (+0.0)	41.7 (+0.0)	41.8 (+0.0)	41.9 (+0.2)
AMA_COL	20.2 (+0.0)	20.2 (+0.0)	20.2 (+0.0)	20.3 (+0.0)	20.2 (+0.0)	20.2 (+0.0)	20.2 (+0.0)	20.2 (+0.0)	20.2 (+0.0)	20.3 (+0.1)
TIE_ARG	11.4 (+0.0)	11.4 (+0.0)	11.4 (+0.0)	11.4 (+0.0)	11.4 (+0.0)	11.4 (+0.0)	11.4 (+0.0)	11.4 (+0.0)	11.4 (+0.0)	11.4 (+0.0)
NSA_NSA	13.8 (+0.0)	13.8 (+0.0)	13.8 (+0.0)	13.8 (+0.0)	13.8 (+0.0)	13.7 (-0.1)	13.8 (+0.0)	13.7 (-0.1)	13.8 (+0.0)	13.9 (+0.0)
ORI_COL	29.9 (+0.0)	29.9 (+0.0)	29.9 (+0.0)	29.9 (+0.0)	29.9 (+0.0)	29.8 (-0.1)	29.9 (+0.0)	29.8 (-0.1)	29.9 (+0.0)	29.9 (+0.0)
NWS_ECU	44.1 (-0.1)	43.7 (-0.5)	44.1 (-0.1)	43.8 (-0.4)	44.1 (-0.1)	43.5 (-0.7)	44.1 (-0.1)	43.5 (-0.6)	44.1 (-0.1)	43.9 (-0.3)

Note: Values show the percentage of species being threatend or endangered of extinction in the year 2050 in each FPU under different scenarios. The value in parentheses give the percentage point change compared to 2010 given different crop production and cropland expansion pathways. The lower bound reflects a risk of biodiversity loss if 100% of crop land expands over existing pasture land, while the upper bound reflects a risk of biodiversity loss if 100% of crop land expands over natural vegetation. Considered crops in the analysis are maize, rice, wheat, soybeans, sugarcane, potatoes, sorghum. FPU = Food Producing Unit. To locate Food Producing Units see S1 Figure and S1 Table. BAU refers to the Business-as-Usual scenario. Scenarios are described in Table 1 in the main text. Those FPUs are listed first that show the highest increase in risk of biodiversity loss according to the upper bound land expansion pathway under the BAU scenario.
