# Supplementary material for: The Role of Latin America’s Land and Water Resources for Global Food Security: Environmental Trade-Offs of Future Food Production Pathways
Source: PLoS One. 2015 Jan 24;10(1):e0116733. doi: 10.1371/journal.pone.0116733 (PMC4305321; doi:10.1371/journal.pone.0116733)
Supplement: S8 Table — (PDF) [file pone.0116733.s019.pdf]

S8 Table. Species risk of extinction and endangerment due to livestock production across Food Producing Units in Latin America and the Caribbean in 2050 (index in %) and net changes between 2010 and 2050 (in %age points)

FPU	(1) BAU		(1a) BAU liberal		(2) Intensification / (3) Sust.-intens.		(4) Yield gaps closed		(5) Extensification	
**Central America and the Caribbean**										
MIM_MEX	29.5	(+0.6)	29.7	(+0.8)	29.7	(+0.7)	29.7	(+0.7)	30.2	(+1.2)
CAM_CCA	44.3	(+0.0)	44.3	(+0.0)	44.3	(+0.0)	44.3	(+0.0)	44.3	(+0.0)
CAR_CCA	57.7	(+0.0)	57.7	(+0.0)	57.7	(+0.0)	57.7	(+0.0)	57.7	(+0.0)
CUB_CCA	41.4	(+0.0)	41.4	(+0.0)	41.4	(+0.0)	41.4	(+0.0)	41.4	(+0.0)
RIG_MEX	28.5	(+0.0)	28.5	(+0.0)	28.5	(+0.0)	28.5	(+0.0)	28.5	(+0.0)
UME_MEX	28.7	(+0.0)	28.7	(+0.0)	28.7	(+0.0)	28.7	(+0.0)	28.7	(+0.0)
YUC_MEX	33.6	(+0.0)	33.6	(+0.0)	33.6	(+0.0)	33.6	(+0.0)	36.3	(+2.8)
**South America**										
TOC_BRA	52.2	(+6.5)	52.2	(+6.5)	52.2	(+6.4)	52.2	(+6.5)	54.3	(+8.5)
ORI_NSA	41.6	(+5.9)	43.4	(+7.7)	43.1	(+7.4)	43.1	(+7.4)	46.2	(+10.5)
AMA_ECU	33.0	(+5.6)	34.4	(+7.0)	34.1	(+6.7)	34.1	(+6.7)	36.9	(+9.5)
PAR_CSA	42.3	(+4.9)	43.1	(+5.7)	43.0	(+5.6)	43.0	(+5.6)	45.1	(+7.7)
AMA_CSA	30.8	(+4.1)	32.1	(+5.4)	31.9	(+5.2)	31.9	(+5.2)	34.1	(+7.3)
AMA_PER	21.6	(+2.8)	22.5	(+3.6)	22.3	(+3.5)	22.3	(+3.5)	23.7	(+4.9)
AMA_COL	22.7	(+2.5)	22.8	(+2.6)	22.7	(+2.5)	22.7	(+2.5)	23.6	(+3.4)
NSA_NSA	16.0	(+2.2)	16.7	(+2.8)	16.5	(+2.7)	16.5	(+2.7)	17.7	(+3.9)
PAR_ARG	47.6	(+1.3)	48.5	(+2.1)	48.2	(+1.8)	48.2	(+1.8)	49.5	(+3.1)
CHC_CHL	25.8	(+0.5)	26.9	(+1.7)	26.8	(+1.5)	26.8	(+1.5)	28.1	(+2.9)
AMA_BRA	20.1	(+0.0)	20.4	(+0.3)	20.2	(+0.1)	20.2	(+0.1)	20.9	(+0.8)
NEB_BRA	48.6	(+0.0)	48.6	(+0.0)	48.6	(+0.0)	48.6	(+0.0)	48.6	(+0.0)
NWS_ECU	44.2	(+0.0)	44.2	(+0.0)	44.2	(+0.0)	44.2	(+0.0)	53.8	(+9.6)
ORI_COL	29.9	(+0.0)	29.9	(+0.0)	29.9	(+0.0)	29.9	(+0.0)	29.9	(+0.0)
PAR_BRA	57.5	(+0.0)	56.5	(-1.0)	56.1	(-1.4)	56.1	(-1.4)	57.7	(+0.1)
PEC_PER	20.5	(+0.0)	20.5	(+0.0)	20.5	(+0.0)	20.5	(+0.0)	20.5	(+0.0)
RIC_ARG	16.1	(+0.0)	16.1	(+0.0)	16.1	(+0.0)	16.1	(+0.0)	16.1	(+0.0)
SAL_ARG	48.5	(+0.0)	48.5	(+0.0)	48.5	(+0.0)	48.5	(+0.0)	48.5	(+0.0)
SAN_BRA	52.6	(+0.0)	52.6	(+0.0)	52.6	(+0.0)	52.6	(+0.0)	52.6	(+0.0)
TIE_ARG	11.4	(+0.0)	11.4	(+0.0)	11.4	(+0.0)	11.4	(+0.0)	11.4	(+0.0)
URU_BRA	58.4	(+0.0)	58.4	(+0.0)	58.4	(+0.0)	58.4	(+0.0)	58.4	(+0.0)
NWS_COL	40.1	(-1.7)	41.5	(-0.2)	41.4	(-0.4)	41.3	(-0.4)	42.2	(+0.5)

Note: Values show the percentage of species being threatend or endangered of extinction in the year 2050 in each FPU under different scenarios. The value in parentheses give the percentage point change compared to 2010. It is assumed that pasture land due to increasing livestock production entirely expands over natural vegetation. FPU = Food Producing Unit. To locate Food Producing Units see S1 Figure and S1 Table. BAU refers to the Business-as-Usual scenario. Scenarios are described in Table 1 in the main text. Those FPUs are listed first that show the highest increase in risk of biodiversity loss under the BAU scenario.
